# Mapping intellectual structure and research hotspots in the field of fibroblast-associated DFUs: a bibliometric analysis

**DOI:** 10.3389/fendo.2023.1109456

**Published:** 2023-04-14

**Authors:** Yushu Zhu, Jianyu Lu, Siqiao Wang, Dayuan Xu, Minjuan Wu, Shuyuan Xian, Wei Zhang, Xirui Tong, Yifan Liu, Jie Huang, Luofeng Jiang, Xinya Guo, Sujie Xie, Minyi Gu, Shuxin Jin, Yicheng Ma, Runzhi Huang, Shichu Xiao, Shizhao Ji

**Affiliations:** ^1^ Department of Burn Surgery, The First Affiliated Hospital of Naval Medical University, Shanghai, China; ^2^ Research Unit of Critical Techniques for Treatment of Burns and Combined Burns and Trauma Injury, Chinese Academy of Medical Sciences, Shanghai, China; ^3^ School of Medicine, Tongji University, Shanghai, China; ^4^ School of Medicine, Shanghai Jiao Tong University, Shanghai, China

**Keywords:** diabetic foot ulcers (DFUs), fibroblast, bibliometric analysis, pathophysiological process, therapeutic targets

## Abstract

**Background:**

Diabetic foot ulcers (DFUs) are one of the most popular and severe complications of diabetes. The persistent non-healing of DFUs may eventually contribute to severe complications such as amputation, which presents patients with significant physical and psychological challenges. Fibroblasts are critical cells in wound healing and perform essential roles in all phases of wound healing. In diabetic foot patients, the disruption of fibroblast function exacerbates the non-healing of the wound. This study aimed to summarize the hotspots and evaluate the global research trends on fibroblast-related DFUs through bibliometric analysis.

**Methods:**

Scientific publications on the study of fibroblast-related DFUs from January 1, 2000 to April 27, 2022 were retrieved from the Web of Science Core Collection (WoSCC). Biblioshiny software was primarily performed for the visual analysis of the literature, CiteSpace software and VOSviewer software were used to validate the results.

**Results:**

A total of 479 articles on fibroblast-related DFUs were retrieved. The most published countries, institutions, journals, and authors in this field were the USA, The Chinese University of Hong Kong, Wound Repair and Regeneration, and Seung-Kyu Han. In addition, keyword co-occurrence networks, historical direct citation networks, thematic map, and the trend topics map summarize the research hotspots and trends in this field.

**Conclusion:**

Current studies indicated that research on fibroblast-related DFUs is attracting increasing concern and have clinical implications. The cellular and molecular mechanisms of the DFU pathophysiological process, the molecular mechanisms and therapeutic targets associated with DFUs angiogenesis, and the measures to promote DFUs wound healing are three worthy research hotspots in this field.

## 1 Introduction

Diabetes is a severe long-term disease that significantly impacting the lives of individuals, families, and societies globally (1). Globally, 463 million people are living with diabetes worldwide, and this number is predicted to increase by 25% in 2030 and 51% in 2045 (1). Diabetic foot ulcers (DFUs) are among the most frequent and severe complications of diabetes, which typically occur in response to neuropathy, peripheral vascular disease, and decreased resistance to infection (2). It is reported that the lifetime risk of developing DFUs in people with diabetes is potentially as high as 19-34% (3). DFUs are a primary contributor to hospitalizations and amputations in patients with diabetes, placing a significant demand on healthcare systems. The DFUs market alone is estimated to rise from USD 7.03 billion in 2019 to USD 11.05 billion by 2027 (4). In patients with diabetes, persistent hyperglycemia damages the nerves in the foot and ankle, leading to peripheral neuropathy. Combined with the narrowing of the arteries due to fatty deposits with subsequent decreased perfusion and tissue ischemia, this leads to peripheral arterial disease (5). These complications of diabetes can diminish sensation in the foot, leaving the patients more susceptible to injury and complications from DFUs. As DFUs are consistently non-healing, it may eventually lead to amputation, thus causing tremendous physical and psychological pain to the patient. Current DFUs wound care standards include unloading, infection control, debridement, and dressing coverage. As well as adjunctive therapies used in the event of DFU progression, such as hyperbaric oxygen and negative pressure wound therapy (6). However, although the treatment of DFUs has achieved some benefits, no satisfactory solution has been achieved so far. Many patients still have suffered amputations of lower limbs from further wound deterioration.

The tricky part of DFU treatment is that it is a chronic non-healing wound. The natural wound healing process consists of inflammatory, proliferative, and remodeling phases (7). In contrast, in DFUs, wound repair is stalled in the inflammatory phase, resulting in the inability of the wound to heal appropriately (5). Macrophages play an important role in this process. Several previous studies have shown that excessive activation of the M1 phenotype of macrophages and impaired M1 to M2 conversion are important mechanisms leading to non-healing of DFU wounds (8–10). However, the role of fibroblasts in DFU wounds cannot be ignored. Dermal fibroblasts are the key cells in wound healing (11). Following the end of the inflammatory phase, the fibroblasts migrate to the wounds in response to various cytokines released from the wound surface (12). They contribute dramatically to wound healing and control wound contraction by forming an extracellular matrix and secreting multiple cytokines (13). More importantly, increased apoptosis and functional disruption of fibroblast-related DFUs led to decreased production of cytokines and extracellular matrix and reduced proliferation and migration capacity, thus hindering wound healing. Therefore, insight into research hotspots and development trends in this area is critical to advancing molecular mechanisms of fibroblast-associated DFUs. More importantly, this may contribute to the potential therapeutic goals of accelerating DFU wound healing, avoiding amputation, and preventing DFU recurrence. In recent years, the explosion and popularity of bioinformatics, especially second-generation sequencing technologies and single-cell sequencing, have allowed researchers to study diabetic fibroblasts in depth and detail. Previous studies have shown that compared to normal fibroblasts, diabetic fibroblasts have a decreased ability to produce, assemble and remodel the extracellular matrix (14) and secrete the vascular endothelial growth factor VEGF. In addition, they have inhibited motility, proliferation, migration, and collagen synthesis (15, 16) and advanced cellular senescence (17), as well as alterations in metabolic memory associated with epigenetics (18).

Bibliometrics has become a popular methodology that assists in rapidly identifying research hotspots, trends, and frontiers in a specific research field based on statistics, network structures, and text analytics (19). Recently, it has been used extensively in multiple research areas, such as coronavirus disease, obesity, triple-negative breast cancer (TNBC), and pancreatic cancer (20–22). These contribute substantially to discovering the latest research hotspots and guiding clinical treatment. From among that, they have identified the newest research hotspots on TNBC, such as immunotherapy, targets, PARP inhibitors, TNBC protein, and receptors. They have taken a significant step forward in addressing drug resistance and tolerance issues to finding the best chemotherapy regimen. Although various meta-analyses and systematic reviews have explicitly addressed research on fibroblast-related DFUs, there still needs to be bibliometric research providing the developing trends and research hotspots in this domain. Therefore, we compiled the scientific literature on the study of fibroblast-related DFUs since the 21st century derived from the Web of Science (WoSSC) database. Furthermore, Biblioshiny software, VOSviewer, and CiteSpace were used to visually analyze the retrieved literature to identify research hotspots and trends in this field (23–25). As there is no comparable bibliometric analysis of research on fibroblast-related DFUs, our work provided a research foundation, frontiers, future trends, and future research hotspots in this field.

## 2 Materials and methods

### 2.1 Search strategy

The Web of Science (WOS) is the greatest global database for collecting and retrieving publications from multiple academic disciplines. Searches were performed based on the WOS Core Collection (WOSCC) database to obtain literature in the Science Citation Index Expanded (SCI-EXPANDED) on April 27^th^, 2022. The literature retrieval strategy was as follows. literature type=article, year=2000-2022. (((TS=diabetic foot ulcer) OR (TS= diabetic foot)) AND ((TS= fibroblast) OR (TS=fibroblasts))). After excluding literature that did not meet the language and article type requirements, we selected the rest of the literature by assessing the title and abstract of articles to determine whether they should be included or excluded. The raw data can be found in **Supplementary Material** (**Supplemental File 1**). To avoid frequent database update bias, all literature searches and data extractions were performed on April 27^th^, and all results were imported into Bibliometrics analysis tools for further analysis.

### 2.2 Data analysis

VOSviewer (24) and Citespace (25) are tools commonly used in knowledge mapping and visualization analysis of scientific literature. Bibliometrix is an open-source tool for performing bibliometric analysis, comprehensive visualization, and knowledge mapping analysis (23). The original data retrieved from WOSCC were analyzed using the bibliometrix package in R version 4.2.0 (Institute for Statistics and Mathematics, Vienna, Austria; www.r-project.org). Biblioshiny software was primarily performed to visualize all retrieved literature and generate visual maps. A visual analysis of annual scientific output and average citation counts provides access to trends in the field. The impact of countries, institutions, authors, and journals is estimated through visual analysis of various bibliometric indicators such as production, citation counts, and H-index. H-index is commonly utilized to evaluate a scholar’s scientific influence and outputs concisely and usefully. Inter-country and inter-author collaboration analyses were also performed, and country collaboration network and author collaboration network maps were generated. Subsequently, high-frequency keywords and highly cited literature analyses were performed. A keyword clustering network map and a historical direct citation network map were constructed to summarize the research hotspots in the field. Based on the analysis of the thematic map, trend topics map, and historical direct citation network map, we outlined the research frontiers and development of fibroblast-related DFUs. CiteSpace software version 6.1.2R was also performed to validate the analysis results (25).

## 3 Results

### 3.1 The growth of fibroblast-related DFUs is steadily increasing and arousing increasing concern

The total number of publications (NP) over a given period could quantitatively and objectively reflect the general development trend of a specific field. A total of 479 articles on fibroblast-related DFUs were published in the WOSCC from January 1^st^, 2000 to April 27^th^, 2022. The annual publications and the average number of annual citations are presented in **Figures S1A**, **S1B**. The overall trend in the number of documents related to fibroblast-related DFUs has gradually increased since 2000, despite some fluctuations during this period. The growth has been rapid since 2011 and maintained a high level after 2016. Additionally, the number of annual citations is increasing rapidly. These findings generally indicated that the research on fibroblast-related DFUs has gradually stabilized. It also meant that fibroblast-related DFUs are arousing growing concern and have significant clinical significance and potential for essential experimental development.

### 3.2 The USA and China were the most two influential and contributing countries in fibroblast-related DFUs research

**Figure 1:**
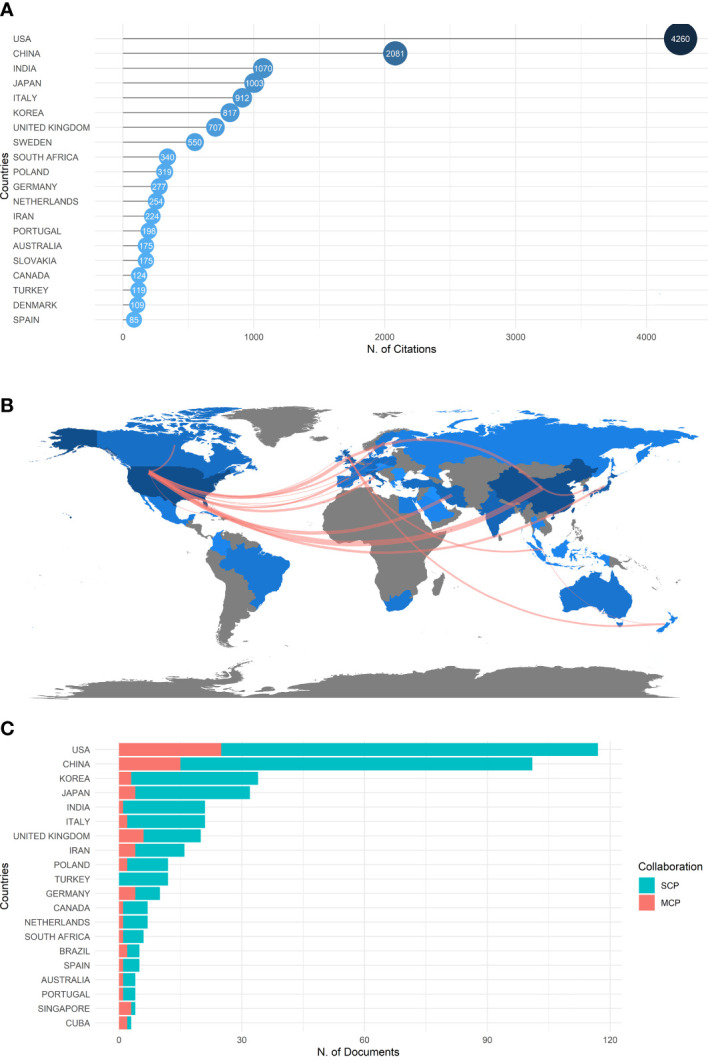
Central countries/regions of fibroblast-related DFUs research production and collaboration. The USA and China were the most two influential and contributing countries in fibroblast-related DFUs research. **(A)** The top 20 countries/regions of fibroblast-related DFUs research with the highest number of publications. **(B)** Countries/Regions production and collaboration world map of fibroblast-related DFUs research. **(C)** Single-country and multi-country publications for the top 20 most productive countries/regions.

The country scientific production map showed the distribution and numbers of publications by countries/regions worldwide (**Figure S2A**). The USA had the most publications (n=441), and its total citation is 4260 (**Figure 1A**), followed by China (321 records cited 2081 times) and Japan (116 records cited 1070 times). This indicated that the USA had the highest publication production and citations and is the leading prolific and impactful country for fibroblast-related DFUs research. According to the visualization country cooperation map (**Figure 1B**), the USA had the most significant central connection point, which indicated that the USA had the most collaborations with other publishing countries. While the line between the USA and China was the widest, it was noted that these two countries collaborated closely on fibroblast-related DFUs research. In contrast, the strength of research and inter-country partnerships in other countries can be further developed. Besides, **Table 1** and **Figure 1C** illustrate the number of single-country and multi-country publications for the top 20 most productive countries/regions.

**Table 1:** Top 20 most productive countries/regions for fibroblast-related DFUs research.

Rank	Country	Publications	Proportion of Publications (%)	SCP	MCP	Proportion of MCP (%)
1	USA	117	24.48%	92	25	21.37%
2	China	101	21.13%	86	15	14.85%
3	Korea	34	7.11%	31	3	8.82%
4	Japan	32	6.70%	28	4	12.50%
5	India	21	4.39%	20	1	4.76%
6	Italy	21	4.39%	19	2	9.52%
7	United Kingdom	20	4.18%	14	6	30.00%
8	Iran	16	3.35%	12	4	25.00%
9	Poland	12	2.51%	10	2	16.67%
10	Turkey	12	2.51%	12	0	0.00%
11	Germany	10	2.09%	6	4	40.00%
12	Canada	7	1.46%	6	1	14.29%
13	Netherlands	7	1.46%	6	1	14.29%
14	South Africa	6	1.26%	5	1	16.67%
15	Brazil	5	1.05%	3	2	40.00%
16	Spain	5	1.05%	4	1	20.00%
17	Australia	4	0.84%	3	1	25.00%
18	Portugal	4	0.84%	3	1	25.00%
19	Singapore	4	0.84%	1	3	75.00%
20	Cuba	3	0.63%	1	2	66.67%

SCP, single-country publications; MCP, multiple-country publications.

All institutions involved in the fibroblast-related DFUs were ranked based on the number of publications. **Figure S2B** shows the top 20 institutions with 326 relevant publications. The Chinese University of Hong Kong published the maximum number of publications (n=39), followed by Harvard University (n=59) and Shahid Beheshti University Medical Sciences (n=52). These three institutions are from China, the USA, and Iran. In summary, we hypothesized that the USA and China were the two most influential and contributing countries in fibroblast-related DFUs research.

### 3.3 The journal of wound repair and regeneration was a critical pathway to access the research frontiers and crucial information of fibroblast-related DFUs research

**Figure 2:**
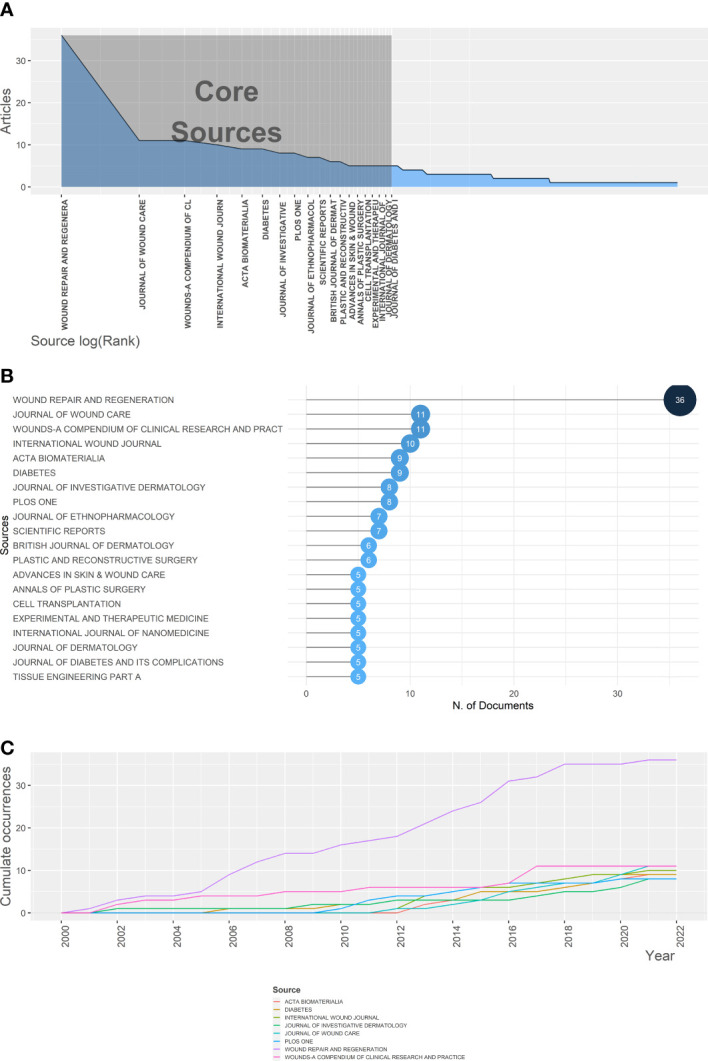
The Journal of Wound Repair and Regeneration was a critical pathway to access the research frontiers and crucial information of fibroblast-related DFUs research. **(A)** Core journals of fibroblast-related DFUs research based on Bradford’s Law. **(B)** The top 20 journals on fibroblast-related DFUs research with the highest number of publications. **(C)** The six highest yielding journals growth of fibroblast-related DFUs research from 2000 to 2022.

Since 2000, 243 sources have published articles on fibroblast-related DFUs research. Based on Bradford’s Law, 19 high-production journals were classified as core sources based on the number of publications (**Figure 2A**) (26). The total number of articles published in the top 20 academic journals is 168 (**Figure 2B**), with 326 total citations (**Figure S2C**). The academic journal Wound Repair and Regeneration published the maximum number of articles (n=36), and its full citation is 664. Followed by the Journal of Wound Care (11 records cited 119 times), Wounds A Compendium of Clinical Research & Practice (11 records), International Wound Journal (10 records cited 156 times), and Acta Biomaterialia (9 records cited 139 times). These productive journals are essential sources of knowledge in this field. Wound Repair and Regeneration has the most publications and total citations, indicating its significant impact on fibroblast-related DFUs. Following this journal enables more rapid access to the research frontiers and crucial information in this field. Moreover, **Figure 2C** showed the growth in productivity with time for the top six most productive journals, which indicated that the number of publications per period of these journals increased rapidly.

### 3.4 Woo Kyung Kim and Jonathan A. Garlick were the most two influential and contributing countries in fibroblast-related DFUs research

The H index is predominantly used to evaluate the total influential power of a specific author (27). Since 2000, over 2650 authors have participated in publications on fibroblast-related DFUs research, and 15 authors had more than 25 publications. We identified the top 20 most productive authors, with 168 articles accounting for 35.07% of all articles. The top 20 most productive authors, the top 20 most locally cited authors, and the top 20 most locally influential authors measured by the H-index are presented in **Figures S3A–C**. Seung-Kyu Han had the most publications (n=18), total citations (n=74), and H-index (n=12). Woo Kyung Kim and Jonathan A. Garlick were relative leaders in each indicator. These authors contributed significantly and notably impacted fibroblast-related DFUs research. Seung-Kyu Han developed a fresh human fibroblast allograft approach and achieved positive results in clinical studies, laying a solid foundation for subsequent research (28). Additionally, according to the author’s collaboration network map (**Figure S4A**), Jonathan A. Garlick seemed to be the author with the most significant collaborative network. **Figure S4** shows that Lin Yan has been a relatively active author recently.

### 3.5 Analysis of high-frequency keywords and four research hotspots based on the keyword co-occurrence analysis

**Figure 3:**
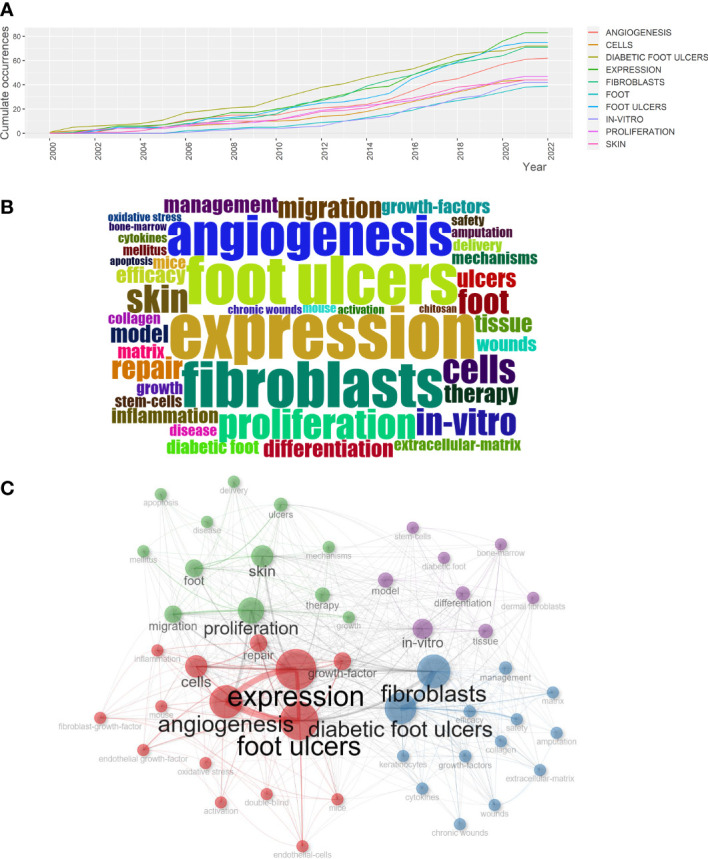
Analysis of high frequency keywords and four research hotspots based on the keyword co-occurrence analysis. **(A)** Top 10 most frequent keywords growth of fibroblast-related DFUs research from 2000 to 2022. **(B)** Visualized word cloud map based on the top 50 most frequent keywords for fibroblast-related DFUs research. **(C)** Visualized keywords co-occurrence network for fibroblast-related DFUs research. Each node indicates a keyword, and the connecting lines between nodes denote the intimacy between keywords. The four clusters were red, blue, green, and purple.

Keywords are highly condensed versions of the critical content of the article and can efficiently identify research hotspots and other significant points (29). **Figure 3A** showed the growth in frequency with time for the top 10 most frequent keywords. It indicated that the keyword “expression” frequency had risen rapidly since 2014, especially after 2019, when it jumped to the number one position. Subsequently, we identified the top 50 high-frequency keywords for fibroblast-related DFUs research with a word cloud (**Figure 3B**) and a tree map (**Figure S5**). Specifically, “expression” had the most frequency of occurrence (n=83), followed by “foot ulcers” (n=76), “diabetic foot ulcers” (n=72), “fibroblasts” (n=72), angiogenesis” (n=63), “proliferation” (n=47), “cells” (n=45), “skin” (n=44), “*in-vitro*” (n=42), and “foot” (n=39). More importantly, Biblioshiny software was performed for keyword co-occurrence analysis and categorized relevant keywords into 4 clusters, thus forming a keyword clustering network map (**Figure 3C**). These clusters reflected the preliminary study content and core research regions to which the keywords referred (30). Within the keyword co-occurrence network graph, each node represents a keyword, and the node size represents the popularity; the line between the nodes indicates the intimacy between the keywords.

Clusters1 (red): Mechanisms of fibroblasts in DFUs pathophysiological process and application of fibroblast-derived related materials. The crucial keywords in this group include “diabetic foot ulcers” (avg. pub. per year as of 2022. 72, 3.27 occurrences), “fibroblasts” (avg. pub. per year as of 2022. 72, 3.27 occurrences), “efficacy” (avg. pub. per year as of 2022. 26, 1.18 occurrences) and “management” (avg. pub. per year as of 2022. 26, 1.18 occurrences).

Cluster2 (blue): The molecular mechanisms and therapeutic targets associated with DFUs angiogenesis. The most recent four hot topics in this cluster include “expression” (avg. pub. per year as of 2022. 83, 3.77 occurrences), “foot ulcers” (avg. pub. per year as of 2022. 76, 3.45 occurrences), “angiogenesis” (avg. pub. per year as of 2022. 63, 2.86 occurrences) and “cells” (avg. pub. per year as of 2022. 45, 2.05 occurrences).

Cluster 3 (green): Bioengineered scaffolds for cutaneous wound healing. The most recent four hot topics in this cluster include “proliferation” (avg. pub. per year as of 2022. 47, 2.14 occurrences), “skin” (avg. pub. per year as of 2022. 44, 2 occurrences), and “foot” (avg. pub. per year as of 2022. 39, 1.77 occurrences), “migration” (avg. pub. per year as of 2022. 32, 1.45 occurrences).

Cluster 4 (purple): Validation of fibroblast differentiation-related mechanisms in DFUs in an *in vitro* model. The most recent four hot topics in this cluster include “*in-vitro*” (avg. pub. per year as of 2022. 42, 2.1 occurrences), “model” (avg. pub. per year as of 2022. 28, 1.27 occurrences), “tissue” (avg. pub. per year as of 2022. 28, 1.27 occurrences) and “differentiation” (avg. pub. per year as of 2022. 27, 1.23 occurrences).

Research on the molecular mechanisms involved in the pathophysiological process of DFUs, the potential therapeutic targets, and the value of bioengineered scaffolds in wound healing in translational medicine have been highly investigated and were the primary research directions.

### 3.6 Relationship between high-impact literature and historical evolution and hotspots

**Figure 4:**
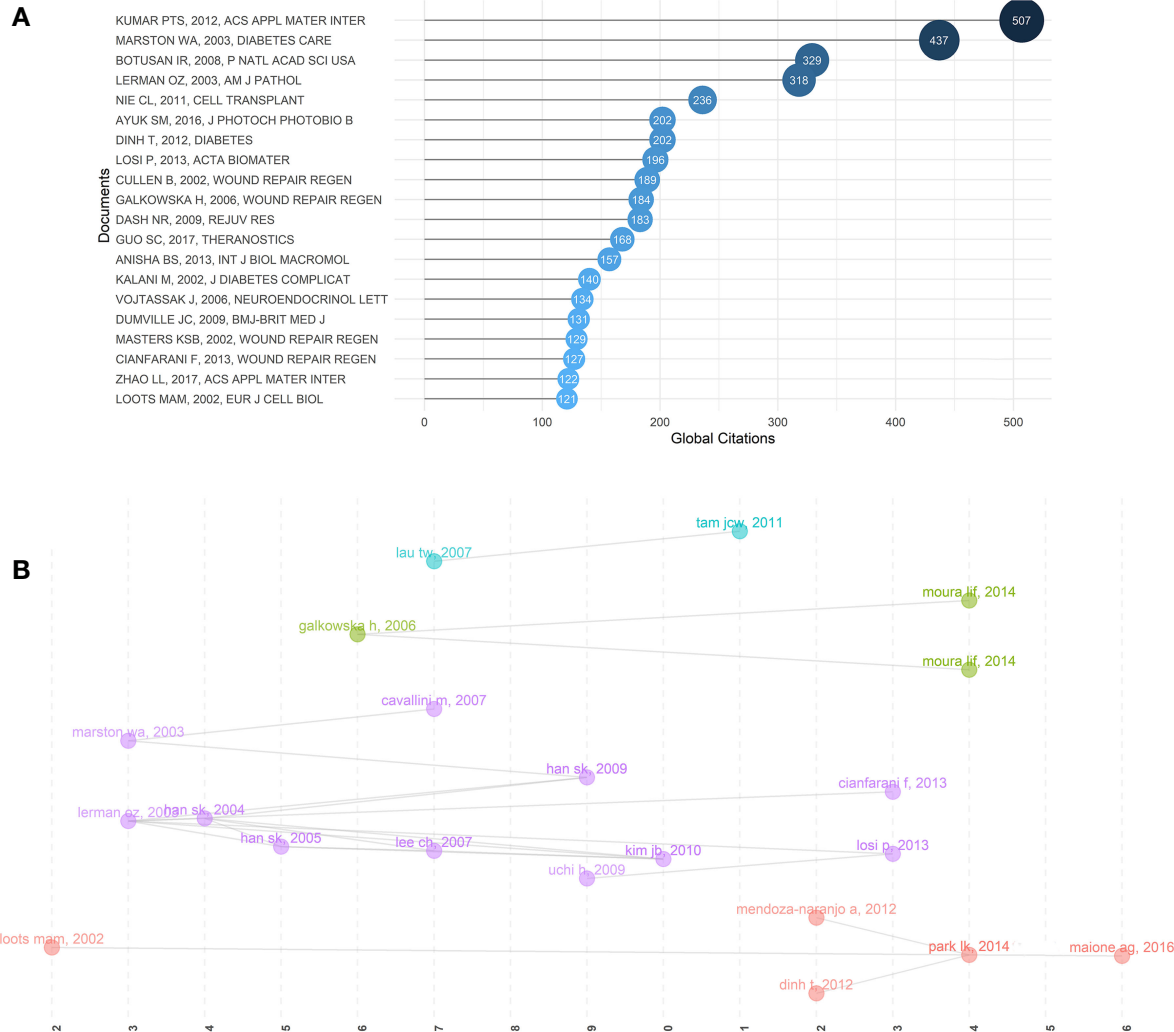
Relationship between high-impact literature and historical evolution and hotspots. **(A)** The top 20 most global cited documents of fibroblast-related DFUs research. **(B)** Visualized historical direct citation network based on the evolution trend of fibroblast-related DFUs research from 2000 to 2022. Each node represents a piece of literature, and the lines between the nodes indicate the citation relationships between publications.

Overall, global citations reflect the impact of an article on the whole database, while local citations reflect the influence of a particular article in our retrieval collection. The top 20 most locally cited documents among the 479 publications were summarized in **Table 2**, along with their journals, authors, and years of publication. **Figure 4A** and **Table 3** showed the top 20 most global cited documents, and 16 documents had more than 130 citations. The article of William A Marston (31) with the title “The efficacy and safety of Dermagraft in improving the healing of chronic diabetic foot ulcers,” which was published in 2003 in Diabetes Care, was the most local cited article (49 citations). Followed by the article with the title “Cellular dysfunction in the diabetic fibroblast: impairment in migration, vascular endothelial growth factor production, and response to hypoxia” by Oren Z. Lerman in 2003 from The American Journal of Pathology with 27 local citations. Then the article “Clinical application of fresh fibroblast allografts for the treatment of diabetic foot ulcers: a pilot study” by Seung-Kyu Han in 2004 from Plastic and Reconstructive Surgery. More importantly, these articles revealed the mechanisms underlying the role of fibroblast dysfunction in non-healing DFUs wounds. The safety and efficacy of human fibroblast-derived dermal substitutes in promoting DFUs healing were demonstrated. A solid foundation has been laid to guide the clinical treatment of complex refractory DFUs.

**Table 2:** Top 20 most local cited documents for fibroblast-related DFUs research.

Rank	Title	Journal	Author	Year	Local Citations	Global Citations	LC/GC Ratio (%)
1	The efficacy and safety of Dermagraft in improving the healing of chronic diabetic foot ulcers: results of a prospective randomized trial.	DIABETES CARE	MARSTON WA	2003	49	437	11.21%
2	Cellular dysfunction in the diabetic fibroblast: impairment in migration, vascular endothelial growth factor production, and response to hypoxia.	AM J PATHOL	LERMAN OZ	2003	27	318	8.49%
3	Clinical application of fresh fibroblast allografts for the treatment of diabetic foot ulcers: a pilot study.	PLAST RECONSTR SURG	HAN SK	2004	21	30	70.00%
4	Chemokines, cytokines, and growth factors in keratinocytes and dermal endothelial cells in the margin of chronic diabetic foot ulcers.	WOUND REPAIR REGEN	GALKOWSKA H	2006	19	184	10.33%
5	Fibroblasts derived from chronic diabetic ulcers differ in their response to stimulation with EGF, IGF-I, bFGF and PDGF-AB compared to controls.	EUR J CELL BIOL	LOOTS MAM	2002	15	121	12.40%
6	Mechanisms involved in the development and healing of diabetic foot ulceration.	DIABETES	DINH T	2012	13	202	6.44%
7	Clinical efficacy of basic fibroblast growth factor (bFGF) for diabetic ulcer.	EUR J DERMATOL	UCHI H	2009	12	111	10.81%
8	Altered ECM deposition by diabetic foot ulcer-derived fibroblasts implicates fibronectin in chronic wound repair.	WOUND REPAIR REGEN	MAIONE AG	2016	10	39	25.64%
9	Potential of human bone marrow stromal cells to accelerate wound healing *in vitro*.	ANN PLAS SURG	HAN SK	2005	9	57	15.79%
10	Efficacy and safety of fresh fibroblast allografts in the treatment of diabetic foot ulcers.	DERMATOL SURG	HAN SK	2009	9	20	45.00%
11	Effect of human bone marrow stromal cells and dermal fibroblasts on collagen synthesis and epithelization.	ANN PLAS SURG	LEE CH	2007	8	12	66.67%
12	Investigation of the effects of Chinese medicine on fibroblast viability: implications in wound healing.	PHYTOTHER RES	LAU TW	2007	7	26	26.92%
13	Effect of human bone marrow stromal cell allograft on proliferation and collagen synthesis of diabetic fibroblasts *in vitro*.	J PLAST RECONSTR AES	KIM JB	2010	7	10	70.00%
14	Fibrin-based scaffold incorporating VEGF- and bFGF-loaded nanoparticles stimulates wound healing in diabetic mice.	ACTA BIOMATER	LOSI P	2013	7	196	3.57%
15	Genome-wide DNA methylation analysis identifies a metabolic memory profile in patient-derived diabetic foot ulcer fibroblasts.	EPIGENETICS-US	PARK LK	2014	7	33	21.21%
16	Autologous fibroblasts to treat deep and complicated leg ulcers in diabetic patients.	WOUND REPAIR REGEN	CAVALLINI M	2007	6	18	33.33%
17	Stabilization of HIF-1alpha is critical to improve wound healing in diabetic mice.	P NATL ACAD SCI USA	BOTUSAN IR	2008	6	329	1.82%
18	The *in vivo* and *in vitro* diabetic wound healing effects of a 2-herb formula and its mechanisms of action.	J ETHNOPHARMACOL	TAM JCW	2011	6	82	7.32%
19	Overexpression of the gap junction protein Cx43 as found in diabetic foot ulcers can retard fibroblast migration.	CELL BIOL INT	MENDOZA-NARANJO A	2012	6	37	16.22%
20	Diabetes impairs adipose tissue-derived stem cell function and efficiency in promoting wound healing.	WOUND REPAIR REGEN	CIANFARANI F	2013	6	127	4.72%

GCs, Global Citations; LCs, local citations.

**Table 3:** Top 20 most global cited documents on fibroblast-related DFUs research.

Rank	TiTle	Author	Journal	Year	TC	TC per Year
1	Flexible and microporous chitosan hydrogel/nano ZnO composite bandages for wound dressing: *in vitro* and *in vivo* evaluation.	KUMAR PTS	ACS APPL MATER INTER	2012	507	46.0909
2	The efficacy and safety of Dermagraft in improving the healing of chronic diabetic foot ulcers: results of a prospective randomized trial.	MARSTON WA	DIABETES CARE	2003	437	21.85
3	Stabilization of HIF-1alpha is critical to improve wound healing in diabetic mice.	BOTUSAN IR	P NATL ACAD SCI USA	2008	329	21.9333
4	Cellular dysfunction in the diabetic fibroblast	LERMAN OZ	AM J PATHOL	2003	318	15.9
5	Locally administered adipose-derived stem cells accelerate wound healing through differentiation and vasculogenesis.	NIE CL	CELL TRANSPLANT	2011	236	19.6667
6	The role of photobiomodulation on gene expression of cell adhesion molecules in diabetic wounded fibroblasts *in vitro*.	AYUK SM	J PHOTOCH PHOTOBIO B	2016	202	28.8571
7	Mechanisms involved in the development and healing of diabetic foot ulceration.	DINH T	DIABETES CARE	2012	202	18.3636
8	Fibrin-based scaffold incorporating VEGF- and bFGF-loaded nanoparticles stimulates wound healing in diabetic mice.	LOSI P	ACTA BIOMATER	2013	196	19.6
9	Mechanism of action of PROMOGRAN, a protease modulating matrix, for the treatment of diabetic foot ulcers.	CULLEN B	WOUND REPAIR REGEN	2002	189	9
10	Chemokines, cytokines, and growth factors in keratinocytes and dermal endothelial cells in the margin of chronic diabetic foot ulcers.	GALKOWSKA H	WOUND REPAIR REGEN	2006	184	10.8235
11	Targeting nonhealing ulcers of lower extremity in human through autologous bone marrow-derived mesenchymal stem cells.	DASH NR	REJUV RES	2009	183	13.0714
12	Exosomes derived from platelet-rich plasma promote the re-epithelization of chronic cutaneous wounds *via* activation of YAP in a diabetic rat model.	GUO SC	THERANOSTICS	2017	168	28
13	Chitosan-hyaluronic acid/nano silver composite sponges for drug resistant bacteria infected diabetic wounds.	ANISHA BS	INT J BIOL MACROMOL	2013	157	15.7
14	Hyperbaric oxygen (HBO) therapy in treatment of diabetic foot ulcers	KALANI M	J DIABETES COMPLICAT	2002	140	6.6667
15	Autologous biograft and mesenchymal stem cells in treatment of the diabetic foot.	VOJTASSAK J	NEUROENDOCRINOL LETT	2006	134	7.8824
16	Larval therapy for leg ulcers (VenUS II): randomised controlled trial.	DUMVILLE JC	BMJ-BRIT MED J	2009	131	9.3571
17	Effects of nitric oxide releasing poly(vinyl alcohol) hydrogel dressings on dermal wound healing in diabetic mice.	MASTERS KSB	WOUND REPAIR REGEN	2002	129	6.1429
18	Diabetes impairs adipose tissue-derived stem cell function and efficiency in promoting wound healing.	CIANFARANI F	WOUND REPAIR REGEN	2013	127	12.7
19	pH and Glucose Dual-Responsive Injectable Hydrogels with Insulin and Fibroblasts as Bioactive Dressings for Diabetic Wound Healing.	ZHAO LL	ACS APPL MATER INTER	2017	122	20.3333
20	Fibroblasts derived from chronic diabetic ulcers differ in their response to stimulation with EGF, IGF-I, bFGF and PDGF-AB compared to controls.	LOOTS MAM	EUR J CELL BIOL	2002	121	5.7619

TCs, total citations.

Subsequently, to acquire the interrelationships between this literature and the historical evolution and hotspots of the field, the software performed the historical direct citation network analysis, and a visual map was generated (**Figure 4B**). Each node represents a piece of literature, and the lines between the nodes indicate the citation relationships between publications. Articles with similar subjects and keywords would be integrated into the same cluster. Moreover, articles with a high normalized local citation score were considered vital documents. Based on these connections, papers were grouped into five clusters representing the four research themes of fibroblast-related DFUs since the 21st century. The first cluster (red) can be traced back to 2002 (32), when scholars, represented by Loots, worked on mechanisms related to DFUs generation and healing in the diabetic microenvironment (32). Including increased expression of Cx43 in DFUs dermal fibroblasts retarded fibroblasts migration (33). Increased inflammatory response, expression of inflammatory factors, and abnormal growth factor levels are the primary factors associated with DFUs failure to heal. Targeting these factors may assist in the management of DFUs (34). The second group (purple) focused on several potential therapeutic measures to promote DFUs wound healing (31, 35–39). Clinical study results demonstrated that human fibroblast cell-derived dermal substitutes and autologous *in vitro* expanded fibroblasts are a safe and effective treatment for DFU (31). Besides, the basic fibroblast growth factor (bFGF) also promotes wound healing in patients with DFU (37).

The third cluster (green) is centralized on the mechanisms of dysfunction of fibroblast migration and release of associated growth factors in DFU and associated therapeutic measures (40–42). Reduced expression of leukocyte chemokines and growth factors at the margins of DFU wounds, resultant angiogenesis in DFU wounds, and impaired fibroblast chemotaxis may explain the poor granulation tissue formation and chronic epithelialization of ulcers (41). Neurotensin-loaded collagen dressings significantly stimulate fibroblast migration and collagen deposition by inhibiting the expression of inflammatory factors, thus promoting DFU wound healing (40, 42). The fourth cluster (blue) is centralized on the potential mechanism of Chinese herbal formula made from the herbs Radix Rehmanniae and Radix Astragali in promoting wound healing in DFU. Chinese herbal formula made from the herbs Radix Rehmanniae and Radix Astragali promotes human fibroblast proliferation and angiogenic and anti-inflammatory effects by increasing fibroblast activity in DFU patients, thereby facilitating the healing of DFU wounds (43, 44). Accordingly, we hypothesized that these research themes might indicate the evolution of research hotspots in the research field of fibroblast-related DFUs.

### 3.7 The research status of various hot topics on fibroblast-related DFUs

**Figure 5:**
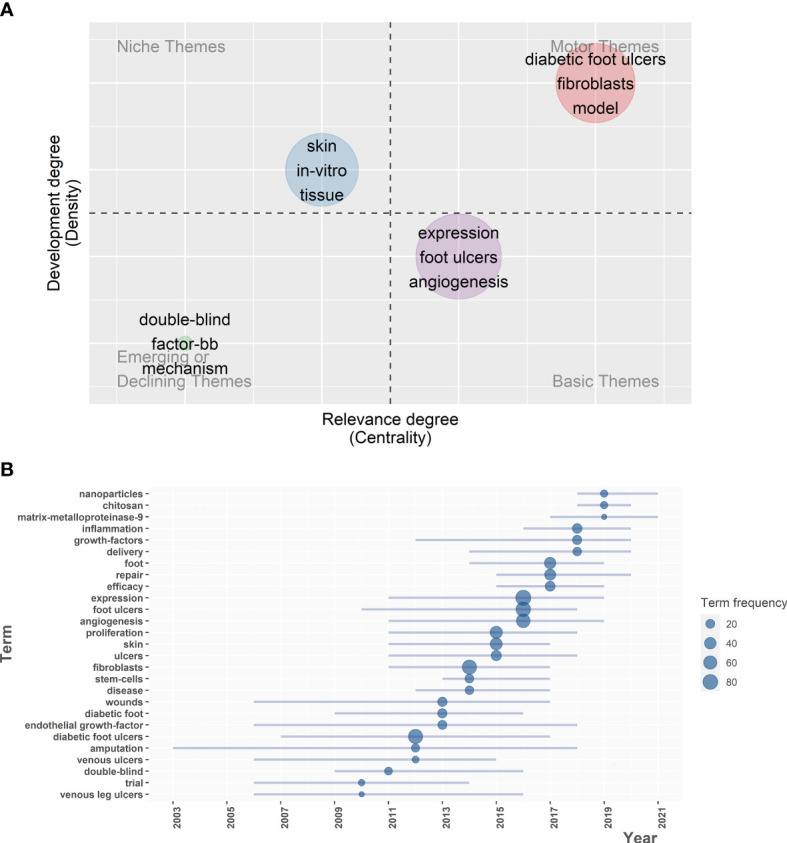
Exploring research status of various hot topics on fibroblast-related DFUs, sketching historical trajectories and revealing research frontiers. **(A)** Thematic map for fibroblast-related DFUs research. The horizontal coordinate refers to the relevance degree (centrality), and the vertical coordinate represents the development degree (density). Motor themes in the first quadrant represents core themes with high centrality and maturity, niche themes in the second quadrant represent isolated themes with increased maturity, the third quadrant represents emerging or declining themes with low centrality and high maturity, and basic themes in the fourth quadrant means popular themes with low maturity. **(B)** Trend topics map for fibroblast-related DFUs research. Showing trends in the occurrence of high frequency keywords for fibroblast-related DFUs research.

Biblioshiny software was performed to construct a two-dimensional thematic map with density as the y-axis and centrality as the x-axis (**Figure 5A**). Density represents the development degree of a single theme, and higher density values mean higher maturity of the theme. Centrality indicates the degree of intimacy with different themes, and high centrality means the heart of the research field.

Motor themes represent the core themes with high centrality and maturity. The crucial keywords in this group include “diabetic foot ulcers”, “fibroblasts”, and “model”. Consistent with clustering 1 in the keyword co-occurrence network, the efficacy of human fibroblast-derived skin substitutes on DFU healing was revealed. Clinical studies have shown that human fibroblast-derived skin substitutes can safely and effectively promote wound healing (45–47). This subject has long been of interest to scholars, and concrete results have been achieved. However, substantial breakthrough research is still urgently needed to drive further developments. Niche themes represent isolated themes with high maturity. The critical keywords in this group include “*in-vitro*”, “skin” and “tissue”. These themes are dedicated to the *in vitro* validation of measures to promote diabetic foot wound healing and related mechanisms. In 1999, the International Diabetic Foot Working Group published an international consensus and guidelines on the management and prevention of diabetic foot, bringing milestones in the management of diabetic foot (48). With the tireless efforts of researchers over the past decades, the physiological knowledge of wound healing and tissue repair, as well as the mechanisms of nonhealing diabetic foot wounds, has become increasingly sophisticated (49–52), which includes an imbalance between the accumulation of ECM components and their remodeling by tissue degrading matrix metalloproteinase(MMPs) (53), reduced or impaired production of growth factors (54, 55), impaired proliferation and migration of keratinocytes and fibroblasts (56). Further, tissue engineering of skin has been developed and extensively studied (57–59). Nevertheless, most of the studies are still in the *in vitro* stage, and high-quality clinical RCT studies may be needed to achieve translation from basic to clinical.

Emerging or declining themes indicate low centrality and maturity themes. The crucial keywords in this group include “double-blind”, “factor-bb”, and “mechanism”. These themes are recommended in the clinical study of human platelet-derived growth factor-BB (becaplermin) in treating patients with DFU. Platelet-derived growth factor-BB (becaplermin) is the most studied growth factor, and its local application in DFU has shown some success. Back in 1998, researchers conducted a phase III randomized, placebo-controlled, double-blind study. It investigated the efficacy and safety of a topical gel formulation of recombinant human platelet-derived growth factor-BB (becaplermin) in patients with chronic neuropathic diabetic ulcers (60). Basic themes represent hot themes with low maturity. The empath keywords in this group include “expression”, “foot ulcers”, and “angiogenesis”. Consistent with clustering 2 in the keyword co-occurrence network, these themes are recommended as the molecular mechanisms and therapeutic targets associated with DFU angiogenesis. With the widespread availability of high-throughput sequencing technology in 2010 and the development of single-cell sequencing technology since 2013, extensive multi-omics and phenotypic correlation studies have been carried out. Consequently, researchers extensively investigated the molecular mechanisms associated with angiogenesis in diabetic foot ulcer wounds and their upstream and downstream potential therapeutic targets. This was a sacred step from basic research to translation to clinical application.

### 3.8 Sketching historical trajectories and exploring research frontiers through trend topics analysis

The trend topics analysis contributes to exploring research hotspots evolution, historical development trajectory, and future research directions in fibroblast-related DFUs. Biblioshiny software was performed to construct the trend topics map (**Figure 5B**). According to **Figure 5B**, it was observed that the evolution of topics related to the research of fibroblast-related DFUs is closely associated with the development of bioinformatics. In patients with diabetic foot, diabetic foot ulcers can be caused by pathogenic factors such as chronic inflammation, peripheral arterial disease, and peripheral neuropathy. However, chronic wound development caused by untreated DFU can often lead to amputation. Hence, Prior to 2010, key topics included “trials”, “venous leg ulcer” and “amputation”, etc. This was probably attributed to the fact that technologies such as high-throughput sequencing were not widespread then, and clinically relevant studies could only be conducted for complications related to diabetic foot ulcers to resolve the patient’s suffering as soon as possible. During this time, researchers have explored multiple pathophysiological mechanisms of DFU trauma and associated therapeutic targets. These include inhibition of fibroblast proliferation migration (61), decreased growth factor release, impaired angiogenesis, disrupted collagen accumulation, and increased levels of MMPs (62). Based on this, various therapeutic measures have been developed and applied to contribute significantly to the clinical management of patients with DFU. These included the use of platelet-derived growth factors, epidermal growth factors (63), inhibition of MMP release (62), and skin substitutes containing components such as collagen and fibroblasts to promote the healing of DFU wounds effectively (53). In contrast, after 2010, the explosion and popularity of second-generation sequencing (64) and single-cell sequencing technologies (64). Scientists turned to study the molecular mechanisms involved in the pathophysiological process of DFU and the potential therapeutic targets. Accordingly, the topic has gradually shifted to “expression”, “angiogenesis”, and “inflammation”, and peaked around 2016, which coincides with the peak in annual publications production.

Additionally, critical topics in recent years were focused on “chitosan”, “nanoparticles”, and “matrix-metalloproteinase-9”. Recent studies have shown that squilla chitosan nanosilver-metal complex and chitosan-hyaluronic acid/nano-silver antimicrobial sponges can be used as potential dressings for wounds infected with DFU-resistant bacteria and effectively inhibit infections with drug-resistant bacteria such as Staphylococcus aureus and Pseudomonas aeruginosa (65, 66). The newly developed chitosan nanoparticle drug delivery system loaded with growth factors and metal oxides effectively promotes the healing of DFU wounds. This includes the removal of pathogens in biofilm structure, a reduced inflammatory response, thorough re-epithelization, and advanced collagen deposition and maturation (67, 68). Therefore, we speculated that applying chitosan-based nanoparticles in DFUs might be a trending topic in the future.

## 4 Discussion

In the present study, we analyzed publications on the research of fibroblast-related DFUs from January 1, 2000 to April 27, 2022 with an information visualization approach. A total of 479 relevant articles were retrieved. The results showed that the trend of publications on the study of this field has continued to grow over time worldwide, exposing that fibroblast-related DFUs have attracted widespread attention from researchers and provided a rich basis for subsequent analyses. The top three countries with a high number of publications and citations were the United States, China, and Japan. Wound Repair and Regeneration, Journal of Wound Care, and Wounds A Compendium of Clinical Research & Practice are the top three most prolific journals. Seung-Kyu Han, Woo Kyung Kim, and Jonathan A. Garlick are the most influential authors with significant status in the field. Subsequently, Biblioshiny software was performed to analyze high-frequency keywords, highly cited documents, and keyword co-occurrence networks. Combined with the results of these analyses, we identified three research hotspots in the fibroblast-related DFUs: the cellular and molecular mechanisms of DFU pathophysiological process, molecular mechanisms and therapeutic targets associated with DFU angiogenesis, and the measures to promote DFUs wound healing. Additionally, bioengineered scaffolds for promoting DFU wound healing are potential directions for researchers to focus on. According to the National Science Foundation Workshop, scaffolds are the ideal resource for repairing, maintaining, and facilitating tissue function (69). Multiple scaffolds have recently been developed as potential materials to promote skin tissue healing (70). Among them mainly included decellularized scaffolds with collagen-rich matrices, microsphere scaffolds composed of a variety of natural polymers, hydrogel scaffolds made up of naturally derived macromolecules or synthetic polymers, and porous scaffolds composed of nanofibers (71–75). Play a unique role in tissue repair and regeneration by providing a suitable platform for supplying various factors associated with cell proliferation and differentiation (75, 76).

Subsequently, the analysis combines a historical direct citation network, a thematic map, and trend topics map. We analyzed the evolution of research hotspots in the field and speculated that applying chitosan-based nanoparticles in DFUs might be a future research direction. In a particular bibliometric analysis, keyword analysis is one of the most indispensable parts, which reflects the general contents and themes of a specific article and represents the research hotspots. The keywords’ variation over time shows the evolution of the field. The research hotspots in the area of fibroblast-related DFUs were summarized as follows.

### 4.1 Cellular and molecular mechanisms of DFUs pathophysiological process

**Figure 6:**
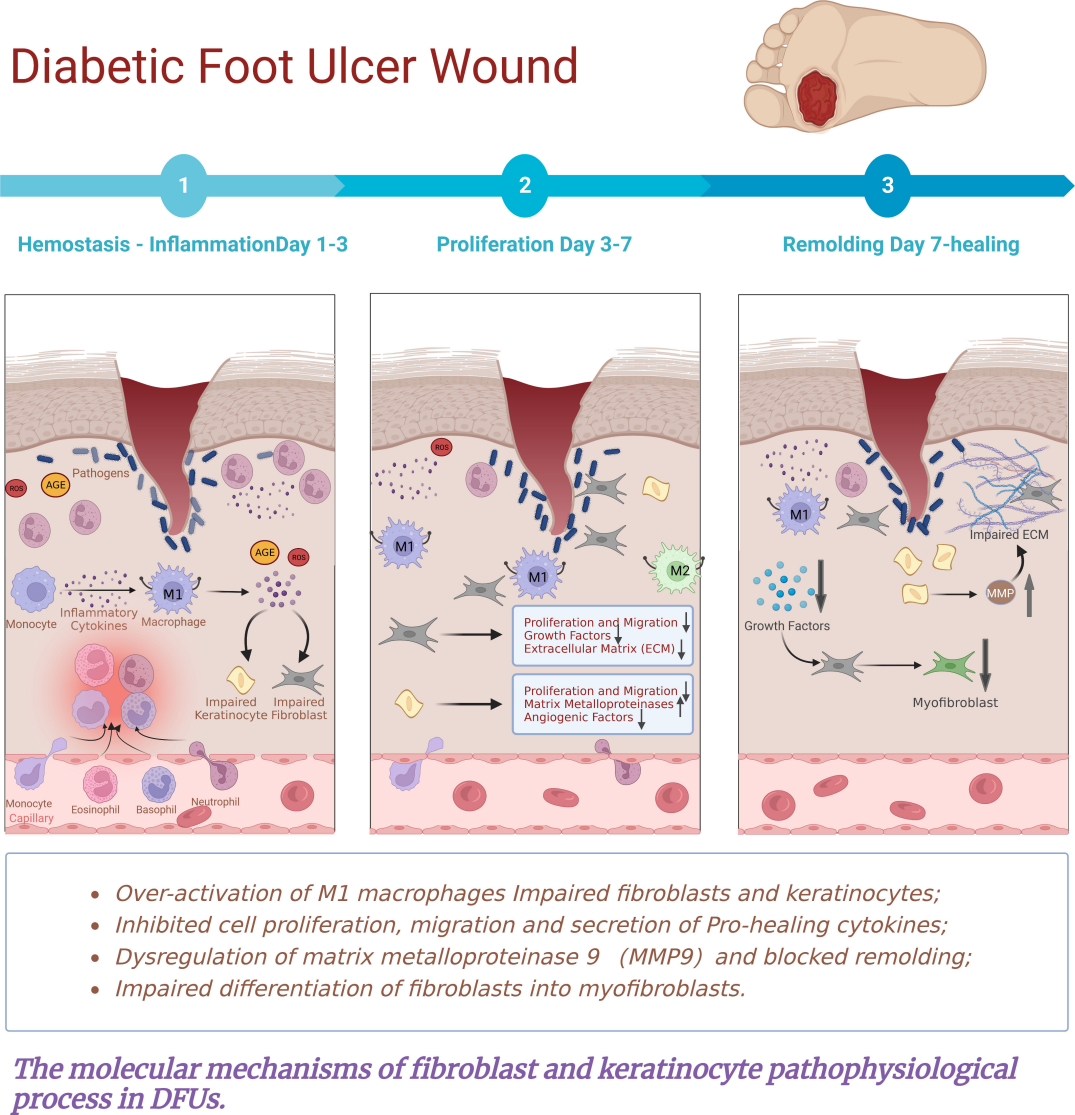
The molecular mechanisms of fibroblast and keratinocyte pathophysiological process in DFUs.The persistent non-healing of DFU wounds is the result of a combination of factors leading to a constant and excessive chronic inflammatory response. In the microenvironment of diabetic wounds, perturbations are associated with hyperglycaemia, advanced glycation end products, oxidative stress and impaired angiogenesis. These factors comprise impaired fibroblasts and disruption of their proliferation, migration, secretion of extracellular matrix and differentiation into myofibroblasts. Meanwhile, there is keratinocyte migration and proliferation, reduced angiogenesis, chronic Inflammation, and abnormal expression of MMPs. Resulting in a constant and excessive chronic inflammatory response, disrupting epithelial cell formation and eventual wound closure. Ultimately leading to the development of chronic non-healing wounds.

**Figure 7:**
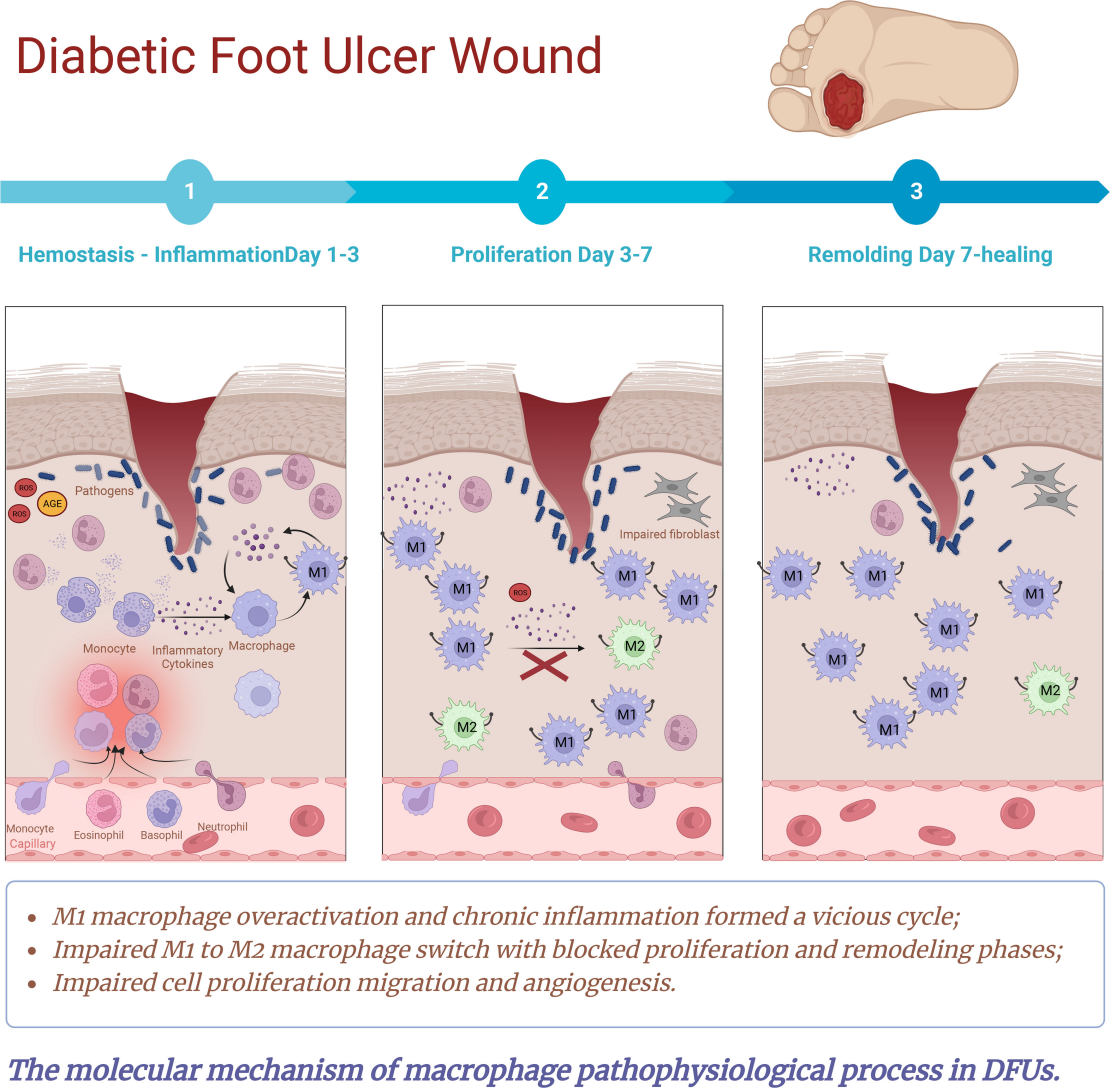
The molecular mechanisms of macrophage pathophysiological process in DFUs. Macrophages play a crucial role in routine wound healing, promoting angiogenesis, collagen deposition and wound closure. Over-activation of M1 macrophages and impaired transition from M1 to M2 phenotype are essential differences between normal and diabetic wound healing. In normal wounds, macrophages clear pathogens and cellular debris by activating a pro-inflammatory phenotype. As the inflammatory phase progresses, macrophages shift from pro-inflammatory phenotype to pro-repair phenotype. As a result, it stimulates the proliferation, differentiation and migration of keratinocytes, fibroblasts and endothelial cells by secreting cytokines and growth factors, which directly or indirectly regulate the proliferative phase of the repair process. However, in the microenvironment of diabetic wounds, perturbations associated with hyperglycaemia, advanced glycation end products, oxidative stress and impaired angiogenesis induce disturbances in the immune microenvironment of diabetic wounds, leading to phenotypic dysregulation as well as quantitative, functional, and epigenetic alterations in traumatic macrophages. As a result, M1 polarization is enhanced and the switch from M1 to M2 is severely impaired. This culminates in a situation where lower numbers of M2 macrophages and higher M1/M2 ratios release low levels of growth factors. Meanwhile, the diabetic microenvironment resulted in macrophage sensitivity to pro-inflammatory cytokines and stimulated macrophages to secrete pro-inflammatory cytokines such as IL-1, IL-6, MMP9, and TNF-a. This further exacerbates the vicious cycle of M1 macrophage polarization and chronic inflammation, causing stagnation of the inflammatory phase. Which created an excess inflammatory cytokines microenvironment, ultimately contributed to impaired fibroblast and keratinocyte migration and delayed wound healing.

DFU is one of the most popular and severe complications of diabetes. The development of DFUs typically occurs in response to neuropathy, peripheral vascular disease, and decreased resistance to infection (2). The persistent non-healing of DFUs wounds may eventually evolve into serious complications such as amputation, causing significant physical and psychological damage to the patient. Thus, an adequate understanding of the mechanisms of functional alterations of DFUs is essential for finding relevant therapeutic targets to promote the healing of diabetic foot ulcers. Cutaneous wound healing is a complex time-dependent multicellular process separated into three overlapping phases: inflammation, proliferation, and remodeling. During this process, multiple cells in the skin, including fibroblasts, keratocytes, and macrophages play essential roles. However, in the diabetic foot wound microenvironment, the normal progression of these phases is impeded, and cellular functions are altered, contributing to a persistent inflammatory state and dysfunctional epithelialization of the wound, ultimately leading to chronic wounds **Figures 6**, **7**.

#### 4.1.1 Fibroblasts and keratocytes

As early as 1977, Rowe’s laboratory (77) pioneered an *in vitro* model of diabetic fibroblasts and demonstrated reduced synthesis, proliferation, and secretion of skin fibroblasts in diabetic patients. This established a solid foundation for the study of fibroblast-related DFUs. Loots’ (61) study showed similar results with reduced proliferative capacity and abnormal morphology of diabetic ulcer fibroblasts compared to the control group. In subsequent studies, the team further discovered that the diabetic environment reduced the ability of fibroblasts to respond to growth factors such as platelet-derived growth factor (PDGF), resulting in abnormal fibroblast function and delayed wound healing. Accordingly, the combination of growth factor PDGF-AB and insulin-like growth factor (IGF-I) treatment may promote diabetic wound healing (32). It was suggested that increased expression of Cx43 may be an underlying cause of poor fibroblast migration and reduced healing rates in diabetic ulcers. The gap junction protein Cx43 (Connexin 43) is central in wound healing (78). In the normal acute wound, keratocytes Cx43 typically downregulate within the first 24-48 hours to migrate toward the wound surface and promote healing (79). A tenfold increase in Cx43 expression in human dermal fibroblasts biopsied from DFUs, which retarded the migration of fibroblasts (33). Similarly, Pollok et al. identified that at the edges of diabetic wounds, there was increased expression of CD43, which induced impaired proliferation and migration of fibroblasts and keratinocytes (80). Besides, Xuan et al. discovered that a high glucose environment inhibited the migration of human fibroblasts in wound healing, which was achieved by inhibiting BFGF to regulate JNK phosphorylation (81). Be note, in the diabetic microenvironment, disturbances accompanied by hyperglycemia, oxidative stress, late glycosylation end products, and impaired angiogenesis. It induces impaired polarization of M2 macrophages, contributing to increased secretion of pro-inflammatory cytokines and a significant decrease in the secretion of anti-inflammatory and growth factors (82). This consequently provokes fibroblasts and keratinocyte damage, impaired proliferation and migration capacity, and wound re-epithelialization.

In addition, high levels of pro-inflammatory cytokines increase the production of matrix metalloproteinases (e.g., MMP9) along with the inhibition of matrix metalloproteinase inhibitor expression. This imbalance further exacerbates extracellular matrix degradation and deprives cells of a scaffold for migration (83). Consequently, fibroblast migration, proliferation, and collagen synthesis are impaired, as well as disrupted wound closure.

Beyond the decreased ability of fibroblasts to proliferate and migrate and secrete growth factors, the power of diabetic fibroblasts to synthesize and secrete extracellular matrix is also disrupted. It was revealed by using a three-dimensional self-assembling ECM model that DFUs-derived fibroblasts have a reduced ability to express, produce, and assemble ECM proteins compared to healthy donor-derived fibroblasts, producing a thin, fibronectin-rich matrix that is involved in the non-healing of diabetic foot wounds leading to the development of DFUs (14). Besides, the early or excessive expression of many aging markers in type 2 Diabetes mellitus contributes to the disruption of diabetic fibroblast function. Senescence is a cellular program that instills proliferative stasis associated with morphological changes, metabolic reprogramming, increased autophagy, apoptosis resistance, and epigenetic reprogramming (84–86). Recent studies suggest that hyperglycemia/oxidative stress/mitochondrial and DNA damage may be the main drivers shaping the senescence phenotype. These adverse agents may trigger replicative senescence of fibroblasts and endothelial cells, thereby impeding DFUs wound healing (17). Wilkinson et al. Discovered that in diabetic mouse trauma, reduced polarization of M2 macrophages resulted in the production of a CXCR2-rich senescence-associated phenotype (85). This induces fibrogenic markers in fibroblasts and ultimately accelerates fibroblast senescence. In a recent study, the Notch pathway, negatively correlated with fibroblast activity, was activated in fibroblasts from the diabetic wound. Increased Notch1 activity inhibits fibroblasts’ growth, migration, and differentiation into myofibroblasts (87). Notch1 signaling dictates the plasticity and function of fibroblasts in wound healing and angiogenesis, and intracellular Notch1 signaling in fibroblasts may represent a potential target for therapeutic intervention in diabetic wound healing (87).

What’s more, various dysfunctions of keratocytes in the DFUs microenvironment have been suggested to be a key factor in non-healing wounds (88). These factors comprise impaired keratinocyte migration and proliferation, reduced angiogenesis, chronic inflammation and infections, oxidative stress. As well as gap junction abnormalities, and abnormal expression of MMPs (49, 89, 90). It was observed that in the diabetic environment, the proliferation and migration of keratinocytes are impaired. This appears to be associated with decreased focal adhesion kinase expression (p125FAK), which determines keratinocyte motility. In addition, elevated expression of various connexins seems to be involved in this process (91, 92).

#### 4.1.2 Macrophages

The persistent non-healing of DFU wounds results from a combination of factors leading to a constant and excessive chronic inflammatory response, disrupting epithelial cell formation and eventual wound closure. Several previous studies have shown that macrophages recruited to the wound site are a vital component of the healing process (93, 94). Macrophages have high plasticity and perform critical roles in all phases of wound repair through host defense, cellular regulatory functions, and tissue debridement (95). Elie Metchnikoff, the father of innate and cellular immunity, first discovered Macrophages, for which he was awarded the Nobel Prize in Physiology or Medicine in 1908 (96). He formulated now generally accepted theories related to the phagocytosis of pathogens by phagocytes such as monocytes, macrophages, and neutrophils (97). Subsequently, until the 1990s, numerous researchers devoted themselves to studying the inflammatory induction and effector functions of macrophages. With the demonstration by Stein et al. in 1992 that IL-4 enhances the expression of the mannose receptor in macrophages, the alternative activation M2 macrophage phenotype came into the public eye (98). The extent and source of phenotypic and functional heterogeneity in macrophage populations and the role of tissue microenvironment in differentially regulating macrophage function have also captured the attention of researchers. All these have laid an essential foundation for unraveling the mystery of the mechanisms of macrophage pathophysiology during wound healing.

Meszaros et al. showed that during the early stages of wound healing, macrophage cleared contaminating microorganisms, apoptotic neutrophils, and cellular debris from the wound surface by phagocytosis (99). Based on the LysMCre/DTR transgenic mouse model of diphtheria toxin-induced macrophage depletion, Goren et al. demonstrated that macrophages play a crucial role in routine wound healing, promoting angiogenesis, collagen deposition, and wound closure (100). Traumatic macrophages are mainly derived from skin resident macrophage populations and bone marrow-derived monocytes (101). They have a diverse function in wound healing to ensure routine healing. During the inflammatory phase, macrophages clear pathogens and cellular debris by activating a pro-inflammatory phenotype. As the inflammatory phase progresses, macrophages engulf traumatized apoptotic cells and release chemokines (e.g., CXCL12, etc.) to facilitate the change from a pro-inflammatory to a pro-repair phenotype (102). As a result, it stimulates the proliferation, differentiation, and migration of keratinocytes, fibroblasts and endothelial cells by secreting cytokines and growth factors, which directly or indirectly regulate the proliferative phase of the repair process (103). During the remodeling phase, M2 macrophages can also digest excess ECM and remodel the structure of the wound by secreting proteases (104), thus playing a crucial role in the whole process of wound healing. However, in the microenvironment of diabetic wounds, perturbations associated with hyperglycaemia, advanced glycation end products, oxidative stress and impaired angiogenesis induce disturbances in the immune microenvironment of diabetic wounds, leading to phenotypic dysregulation as well as quantitative, functional, and epigenetic alterations in macrophages (105–107). This is manifested by a persistent chronic inflammatory response to trauma and stagnation of the repair process during the inflammatory phase, ultimately leading to chronic non-healing trauma (106).

Macrophages present highly plastic, characterized by a critical feature of their phenotype that changes in response to changes in the microenvironment. Based on their surface receptor expression, secretion characteristics and function. Macrophages are mainly classified into the classically activated M1 phenotype and the alternatively activated M2 phenotype (96). M1 macrophages are considered pro-inflammatory since they are driven by pro-inflammatory cytokines such as lipopolysaccharide (LPS) and tumor necrosis factor (TNF). They produce pro-inflammatory cytokines, including interleukin (IL)-12 and IL-23, along with reactive oxygen species (ROS) (108). In contrast, non-classical macrophages, also known as M2 macrophages, are regarded as pro-healing or resolving macrophages. They are stimulated by anti-inflammatory cytokines such as IL-4 and IL-10, and release growth factors such as the insulin-like growth factor (IGF) and transforming growth factor (TGF) (108).

Notably, over-activation of M1 macrophages and impaired transition from M1 to M2 phenotype are essential differences between normal and diabetic wound healing (109). In normal wounds, infiltrating monocytes differentiate into classically activated M1 and alternatively activated M2 macrophages. Whereas in diabetic wounds, M1 polarization is enhanced and the switch from M1 to M2 is severely impaired (105, 110). This culminates in a situation where lower numbers of M2 macrophages and higher M1/M2 ratios release low levels of the growth factors EGF, FGF, PDGF, and VEGF, as well as the anti-inflammatory cytokines IL-10, TGF-α and TGF-β, which are key contributors to the proliferation and remodeling phase (8). Notably, the hyperglycaemic environment is thought to be one of the major pathways leading to an increase in pro-inflammatory cytokines. In particular, 13 pro-inflammatory cytokines, including TNF-a, IL-1, and IL-6, which stimulate the overactivation of M1 macrophages, are upregulated in the hyperglycaemic environment (111). Interestingly, the diabetic microenvironment resulted in macrophage sensitivity to pro-inflammatory cytokines and stimulated macrophages to secrete pro-inflammatory cytokines such as IL-1, IL-6, MMP9, and TNF-a (112). This further exacerbates the vicious cycle of M1 macrophage polarization and chronic inflammation (113), causing stagnation of the inflammatory phase. However, beyond the cellular and molecular mechanisms underlying macrophage plasticity, the maturing field of epigenetics has become a new point of focus in the investigations of macrophage-mediated inflammation. Recent evidence points to the role of epigenetics in regulating macrophage function in diabetic wound healing, including DNA methylation of CpG islands and methylation of histone tails. These processes can trigger increased expression of pro-inflammatory cytokines, which in turn promote further polarization of M1 macrophages (114).

Huang et al. demonstrated that the hyperglycemic wound environment had more infiltration of M1 macrophages, which created an excess TNF-α microenvironment that upregulated TIMP1 expression in keratinocytes (8). This ultimately contributed to impaired keratinocyte migration and delayed wound healing (8). Mirza et al. showed that persistent activity of NOD-like receptor protein (NLRP)-3 inflammasomes in diabetic and mouse wounds resulted in a sustained inflammatory response and impaired healing of the wounds. In contrast, inhibition of inflammasome activity in diabetic mice wounds when applied topically promoted wound healing, induced a shift from a pro-inflammatory phenotype to a healing-associated MP phenotype, and increased pro-healing growth factor levels (115). In addition, M1 macrophages in the wound secrete large amounts of proteases such as MMP9 and reduce inhibitors of MMPs; this imbalance further exacerbates extracellular matrix degradation and deprives cells of a scaffold for migration (83, 116). Consequently, this impairs fibroblast migration, proliferation, and collagen synthesis, disrupting wound closure (83, 116). M2 macrophages also play an essential role in wound angiogenesis and can promote wound angiogenesis through macrophage-endothelial cell adhesion paracrine effects mechanisms (117). Gibson et al. have identified reduced VEGFR1 signaling in diabetic wound tissue, which appears to be associated with impaired angiogenesis (118). Unfortunately, in DFUs wounds, there was impaired activation of M2 macrophages and over-activation of the M1 macrophage phenotype, which contributed to impaired angiogenesis.

Moreover, in the DFUs microenvironment, the number of macrophages and functional alterations are also closely associated with wound healing. The number of macrophages is also closely related to diabetes mellitus in wound healing. Barman et al. showed that diabetes induces myeloid preference in bone marrow progenitor cells in the DFUs microenvironment. This was associated with increased macrophage accumulation in wounds and impaired wound healing (119). Using macrophages isolated from diabetic mouse wounds, Savita et al. first demonstrated impaired clearance of apoptotic cells from diabetic wound macrophages, with significant impairment in efferocytosis (9). This resulted in a markedly increased load of apoptotic cells in the wound tissue, high expression of pro-inflammatory factors and low expression of anti-inflammatory cytokines. Ultimately, it prolongs the inflammatory phase and makes wound healing more difficult. Notably, the senescence-associated secretory profile (SASP) is a robust approach whereby a small number of senescent cells in tissues can exert significant local biological effects and is implicated in the pathogenesis of many chronic diseases (120). Based on a diabetic mouse model, Wilkinson et al. found that wound-derived macrophages from diabetic mice exhibited reduced M2 macrophage polarization and the production of CXCR2-rich SASP (85). This induced fibrotic markers of fibroblasts and had the potential to stimulate fibroblast senescence. In addition, wounds in diabetic mice treated with CXCR2 antagonists showed reduced macrophage senescence and local inflammation and promoted wound closure (85). Interestingly, beyond the cellular and molecular mechanisms underlying macrophage plasticity, the maturing field of epigenetics has become a new point of focus in investigating macrophage-mediated inflammation. Recent evidence points to the role of epigenetics in regulating macrophage function in diabetic wound healing, including DNA methylation of CpG islands and methylation of histone tails (114). These processes can trigger increased expression of pro-inflammatory cytokines, which in turn promote further polarization of M1 macrophages.

### 4.2 Molecular mechanisms and therapeutic targets associated with DFUs angiogenesis

In the current study, research on molecular mechanisms and therapeutic targets related to DFUs angiogenesis can be found in the keyword co-occurrence clustering network map, thematic map, and trend topics map. Combining the analysis of highly cited literature and the extensive related literature, we speculate that the study of molecular mechanisms and therapeutic targets related to DFUs angiogenesis is a focus of academic attention and potentially a research trend for the coming period significant for promoting diabetic wound healing. Angiogenesis refers to expanding its vascular branches by sprouting and forming vascular networks, which are essential for embryonic growth, tissue development, and wound healing (121). Inadequate arterial perfusion associated with peripheral arterial disease, along with macrovascular and microvascular disease, has been reported to be responsible for the chronicity of diabetic foot ulcers (6). Moreover, decreased nutrient supply due to poor granulation tissue angiogenesis is closely associated with impaired healing of diabetic ulcers (122). Inadequate blood perfusion combined with impaired angiogenesis complicates tissue repair in diabetes. Consequently, a thorough understanding of this topic can better reveal the molecular events that delay diabetic wound healing and potential molecular targets that promote healing. Thus, the current dilemma of non-healing DFUs and avoiding serious complications such as amputation can be avoided.

We have identified an explosion of studies related to DFUs angiogenesis starting in 2016. This may be attributed to single-cell sequencing being named “Technology of the Year” by Nature Methods in 2013 (123). It opens new perspectives on exploring molecular mechanisms and therapeutic targets related to DFUs angiogenesis. One of the most researched factors is recombinant human PDGF-BB (Becaprine gel), which has become the only growth factor authorized by the FDA for wound treatment (124). Dopamine is a primary central catecholamine neurotransmitter that controls cognition, mood, and movement and regulates cardiovascular, endocrine, renal, gastrointestinal, and immune functions (125–129). It was shown that activation of dopamine D1 receptors in dermal fibroblasts restores their production of vascular endothelial growth factor A *via* the protein kinase A pathway and consequently restores angiogenesis in subsequent diabetic skin wound tissue (130). Fibrocytes are bone marrow-derived hematopoietic stem cells integral to wound healing (131). Fibrocytes have been found to promote wound healing by facilitating cell proliferation, re-epithelialization, and angiogenesis compared to dermal fibroblasts and diabetic mice treated with PBS (132). Furthermore, Xing et al. identified that Netrin-1 levels were lowest in DFUs patients compared to healthy controls and DM patients. In *in vitro* experiments, overexpression of Netrin-1 restored the high glucose-induced impairment of the PI3K/Akt-eNOS pathway *via* restoring NO production that was significantly inhibited by high glucose, thereby improving DFUs angiogenesis (133).

Importantly, with the development of bioinformatics technologies such as high-throughput sequencing in recent years, extensive identification of Long noncoding RNAs (lncRNAs) and microRNAs (miRNAs) by scholars has opened new doors to studying the regulation of gene expression. lncRNAs have been demonstrated to be involved in the abnormal regulation of angiogenic genes by regulating the stability and translation of mRNAs (134). A recent study suggested that lncRNA Metastasis-associated lung adenocarcinoma transcript 1 (MALAT1), which is poorly expressed in DFUs patients and consistent with the expression of angiogenic factors such as nuclear factor erythroid 2-related factor 2 (NRF2), Hypoxia-inducible factor-1α (HIF-1α) and VEGF (135). Indeed, Nrf2 can positively regulate the MALAT1/HIF-1α loop and thus regulate angiogenesis, which may become a new target for treating diabetic wounds in the future (135). miRNAs are non-coding RNAs of approximately 22 nucleotides implicated in various roles in critical phases of inflammation, angiogenesis, epithelialization, and remodeling in diabetic wound healing (136, 137). Increasingly, miRNAs have been found to be associated with diabetic wound angiogenesis. In the latest study, Wang et al. (138) have discovered that, unlike non-DFUs wounds, the circulating exosome miR-181b-5p in the plasma of DFUs patients facilitates cellular senescence and inhibits angiogenesis *via* the NRF2/HO-1 pathway to impede DFUs healing. In further studies, the team used the miR-181b5p inhibitor *in vivo* experiments and found that angiogenesis was promoted, with the consequent restoration of wound healing capacity. Furthermore, other studies have shown that miR-217 (139) and miR23c (140) are overexpressed in DFUs patients and restrain angiogenesis by inhibiting the HIF-1α/VEGF pathway and targeting stromal-cell-derived factor-1 (SDF-1α). More importantly, inhibition of miR-217 and miR23c could facilitate DFUs angiogenesis by upregulating the above-mentioned corresponding pathways, thereby favoring wound healing. Another exciting study used maggot excreta/secretions to stimulate miR18a/19a overexpression. The results revealed that diabetic wound angiogenesis could be promoted by downregulating thrombochondroitin-1 (TSP-1) expression. This protein inhibits angiogenesis, which could be a new target for DFU therapy (141).

### 4.3 Diabetic foot ulcer management

Back in the mid-19th century, the problem of diabetic foot ulcers was first described (142). In 1852, Marchal de Calvi discovered the phenomenon that there was an association between diabetes mellitus and foot gangrene. Subsequently, in 1854, Thomas Hodgkin was similarly aware of the problem. At that time, given the limited development of science and technology, the most popular method of treating ulcers was prolonged bed rest (142). Not until the late 19th century, the genius surgeon Treves provided a landmark contribution to the treatment of the diabetic foot. He established three essential principles for treating foot ulcers: rapid debridement, offloading pressure, and foot care (143). Afterward, these principles led to today’s standard of care for DFUs: surgical debridement, wound off-loading, dressing coverage, and infection control (144), as shown in **Figure S6**. Along with a wide range of adjunctive therapies, such as growth factors, hyperbaric oxygen (HBOT), and negative pressure wound therapy (NPWT) (145). Furthermore, in addition to these measures, multidisciplinary diabetic foot care is becoming a focal point of treatment (146).

The goal of diabetic foot treatment is to achieve tissue healing while maintaining adequate function and weight bearing to bed (145). Debridement is a gold standard in treating DFUs, including removing necrotic and inactivated tissue, surrounding callus, and foreign debris from the wound. This process contributes to the formation and re-epithelialization of granulation tissue, and reduces the pressure in the plantar region of the foot (147). Meanwhile, removing necrotic tissue destroyed the breeding ground and physical barrier for bacterial colonization, which could be instrumental in controlling traumatic infections (148). A 10-year retrospective study of standardized wound care protocols revealed that amputation rates in diabetic foot patients decreased through timely and effective debridement (149). Current debridement modalities consist of surgical (sharp debridement), biological (maggot therapy), enzymatic (clostridial collagenase), autolytic (hydrogel), mechanical (hydro surgery), and ultrasound (150–152). Of note, according to the recommendations of the Wound Healing Society (WHS), sharp debridement is the preferred approach to debridement (153). High plantar pressure has been recognized as a primary factor in the development and poor healing of DFUs. Offloading not only destresses the ulcer site, but also ensures the redistribution of shear forces which is currently an effective strategy for treating DFUs (148). Off-loading can be accomplished through various mechanisms, including shoe modifications, boots, and orthopedic walkers (148). Guidelines published by the International Working Group on the Diabetic Foot (IWGDF) suggest that in the absence of infection or ischemia, non-removable and knee-high devices (total-contact casts or non-removable walkers) are the preferred treatment for neuropathic forefoot or midfoot plantar ulcers (154). Infection is a fundamental cause of DFUs morbidity, hospitalization, impaired healing, and amputation. Compared to uninfected DFUs, infection increases the risk of lower extremity amputation by 50% (155). In DFUs wounds, factors such as stress and pressure, decreased function of immune cells (e.g., macrophages and neutrophils), and ischemia makes them more susceptible to infection. More importantly, in diabetic ulcers, the infection can spread rapidly and induce cellulitis, abscesses and osteomyelitis. It can also lead to life-threatening infectious infections when treatment is delayed (156). Effective control of traumatic infections can be achieved through surgical and pharmacological approaches, which are essential for managing DFUs. Antibiotics are considered the most effective drugs available to fight infections in clinical practice. Nevertheless, the consequent problem of drug-resistant microorganisms is increasing. Fortunately, a variety of new anti-infective measures have been developed and proven to be effective. Fortunately, a variety of new anti-infective measures have been developed and proven to be effective. These include new vehicles for drug delivery systems (e.g., multifunctional nanomaterials) (157) and Bioactive Antimicrobial Peptides (5, 158), which bring hope for the control of DFUs infections.

The dressing provides a protective barrier to the wound, not only preventing bacterial contamination and maintaining a moist environment on the wound surface; it also promotes granulation, angiogenesis, autolysis processes, and rapid migration of epidermal cells at the base of the wound to promote wound healing (159). Recently, an RCT study including 160 patients presented a shred of critical evidence. That is, the combination of recombinant epidermal growth factor and nanosilver dressings can effectively promote DFUs’ wound healing and prevent infection (160). In addition, with the development of bioengineering science and technology, wound dressings can also be used as drug delivery systems to deliver various therapeutic substances (drugs, growth factors, peptides, stem cells, and other bioactive substances) to the wound surface (161), thus promoting wound healing and performing an instrumental role in the treatment of DFUs. Notably, several studies have shown the beneficial effects of hyperbaric oxygen therapy in promoting wound healing in DFUs (162–164). A recent multicenter RCT of 73 patients with chronic DFUs revealed superior efficacy of multimodality cyclical pressure Topical Wound Oxygen compared to standard care alone at 12 weeks and 12 months (164). In the end, the education of patients on meticulous foot care and appropriate foot products through a multidisciplinary approach must be considered (145).

Nevertheless, there are still some limitations to our study. First, we only retrieved publications from the WoSCC database, which may contribute to an imperfect collection of relevant publications. Second, we only accessed publications from January 1^st^, 2000 to April 27^th^, 2022, which would cause the exclusion of some of the most recent findings as this data is continuously updated. Third, some recent critical publications may have yet to receive sufficient attention and thus may not have been explored in depth. Despite these limitations, this study comprehensively reviews the global status and research trends on fibroblast-related DFUs.

## 5 Conclusion

In the present study, we performed an in-depth analysis of research on fibroblast-related DFUs from a bibliometric perspective. Including an exploration of the current knowledge structure, development trends, research hotspots and future directions of the field. The present study indicated that research on fibroblast-related DFUs is growing. The cellular and molecular mechanisms of DFU pathophysiological process, molecular mechanisms and therapeutic targets associated with DFU angiogenesis, and the measures to promote DFUs wound healing are three worthy research hotspots in this field. Further research on these topics could contribute to a complete understanding of the molecular events in the pathophysiological processes of DFUs and the search for potential therapeutic targets, establishing a solid foundation for achieving clinical translation.

## Data availability statement

The original contributions presented in the study are included in the article/**Supplementary Material**. Further inquiries can be directed to the corresponding authors.

## Ethics statement

The study was approved by the Ethics Committee of the First Affiliated Hospital of Naval Medical University.

## Author contributions

Conception/design: YZ, JL, SW, DX, MW, SYX, WZ, XT, YL, JH, LJ, XG, SJX, MG, SXJ, RH, SCX, and SZJ. Collection and/or assembly of data: YZ, JL, SW, DX, MW, SYX, WZ, XT, YL, JH, LJ, XG, SJX, MG, SXJ, RH, SCX, and SZJ. Data analysis and interpretation: YZ, JL, SW, DX, MW, SYX, WZ, XT, YL, JH, LJ, XG, SJX, MG, SXJ, RH, SCX, and SZJ. Manuscript writing: YZ, JL, SW, DX, MW, SYX, WZ, XT, YL, JH, LJ, XG, SJX, MG, SXJ, RH, SCX, and SZJ. Final approval of manuscript: YZ, JL, SW, DX, MW, SYX, WZ, XT, YL, JH, LJ, XG, SJX, MG, SXJ, RH, SCX, and SZJ. All authors contributed to the article and approved the submitted version.
