# Supplementary material for: Mapping intellectual structure and research hotspots in the field of fibroblast-associated DFUs: a bibliometric analysis
Source: Front Endocrinol (Lausanne). 2023 Apr 14;14:1109456. doi: 10.3389/fendo.2023.1109456 (PMC10140415; doi:10.3389/fendo.2023.1109456)
Supplement: Supplementary file 1 [file DataSheet_1.docx]

**Supplementary Figures**


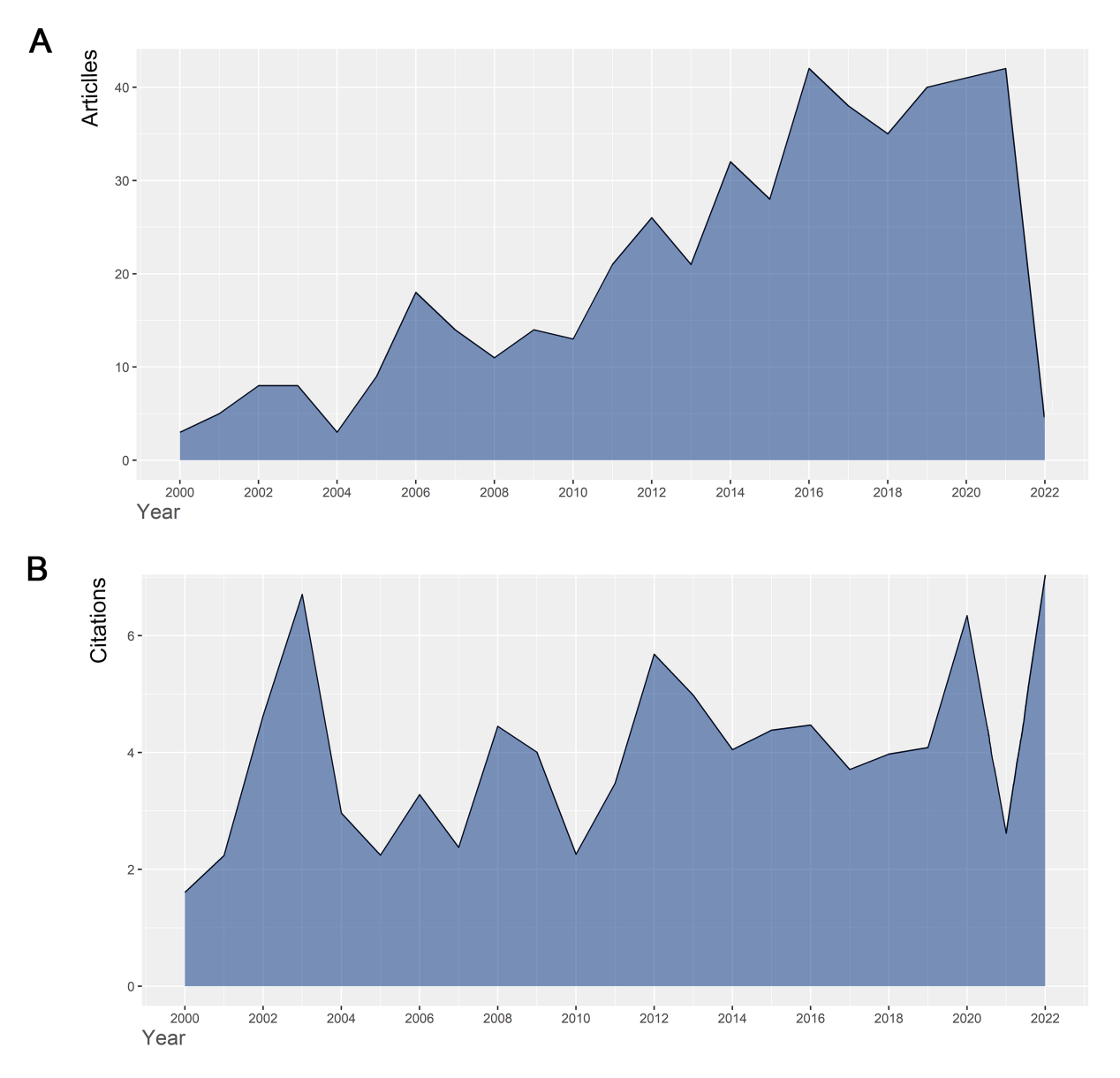


**Figure S1.** The Growth of Fibroblast-Related DFUs is Steadily Increasing and Arousing Increasing Concern.

(A) The growth of fibroblast-related DFUs research was steadily increasing and arousing increasing concern from 2000 to 2022.

(B) The growth of fibroblast-related DFUs research was steadily increasing and arousing increasing concern from 2000 to 2022.

**
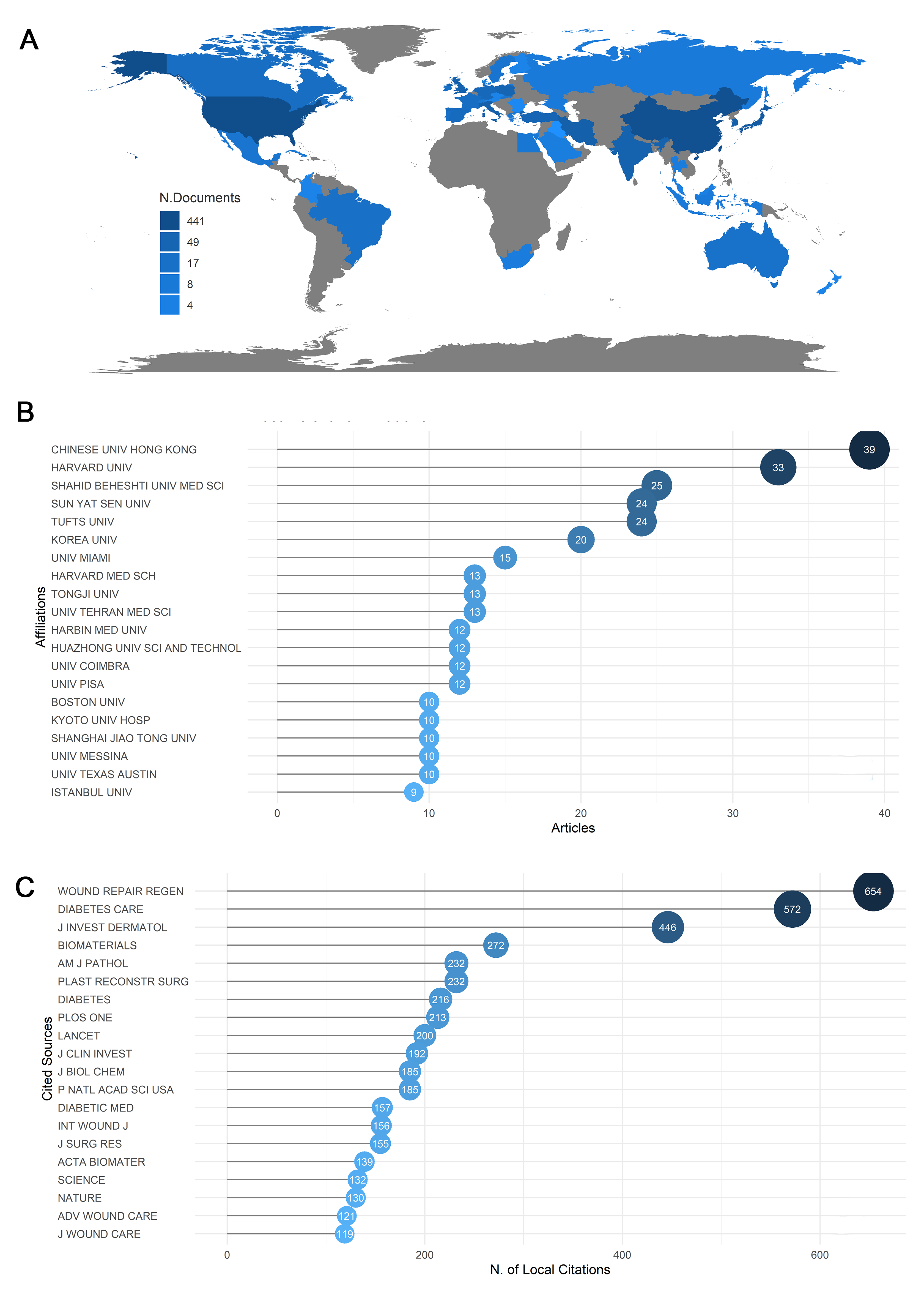
**

**Figure S2.** Supplementary materials for Figure 2 and Figure 3.

1. Countries/Regions distribution world map of fibroblast-related DFUs research.

(B) The top 20 highest producing institutions on fibroblast-related DFUs research.

(C) The top 20 journals on fibroblast-related DFUs research with the highest number of citations.


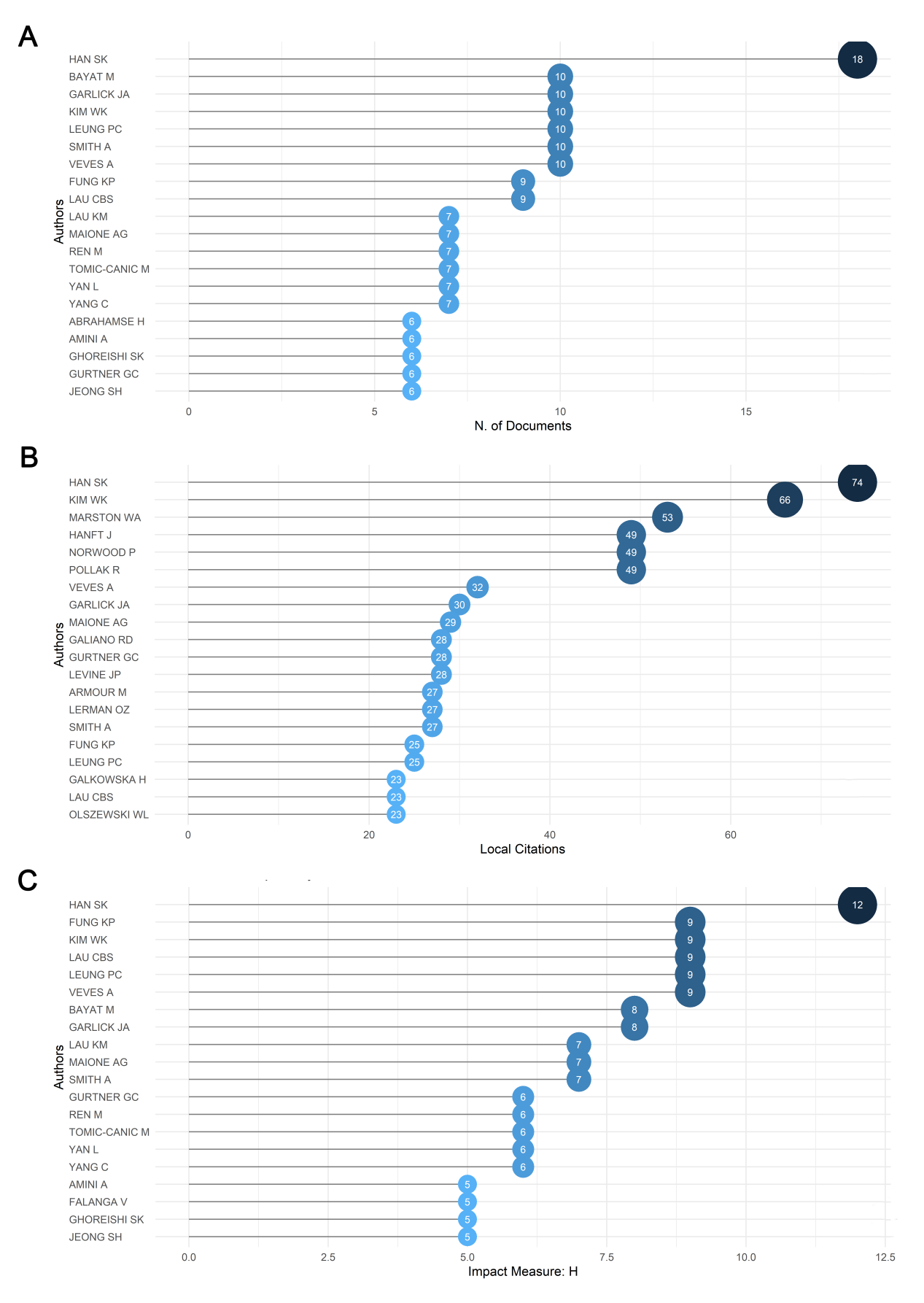


**Figure S3.** Primary authors of fibroblast-related DFUs research production. Woo Kyung Kim and Jonathan A. Garlick were the two most influential and contributing authors in fibroblast-related DFUs research.

(A) The top 20 highest producing authors on fibroblast-related DFUs research.

(B) The top 20 authors on fibroblast-related DFUs research with the highest number of citations.

(C) The top 20 authors on fibroblast-related DFUs research with the highest number of H-index.


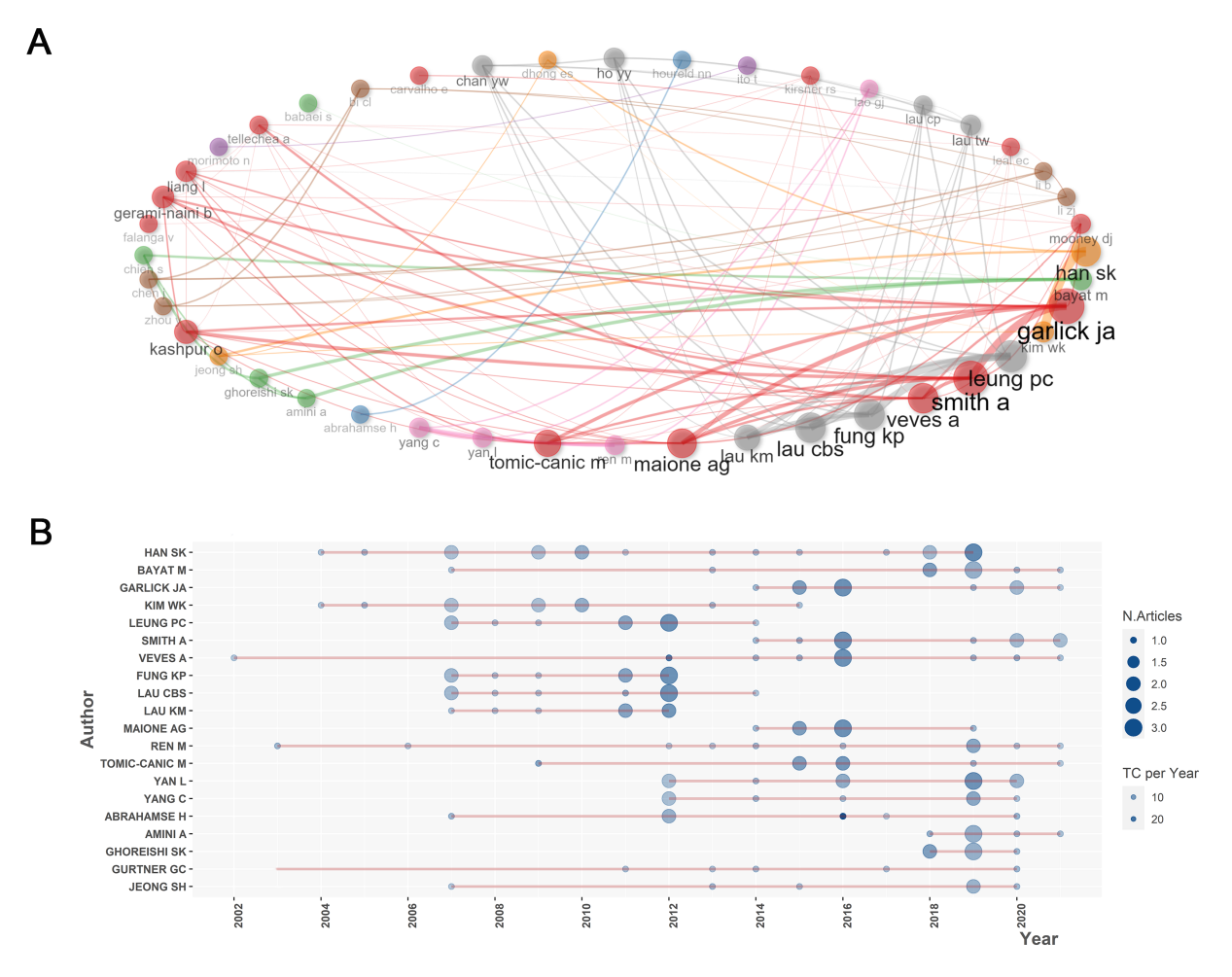


**Figure S4.** Top authors’ production over time and collaboration.

1. A visualization network of collaboration among authors in fibroblast-related DFUs research.

(B) A visualization map of production over time among authors in fibroblast-related DFUs research.


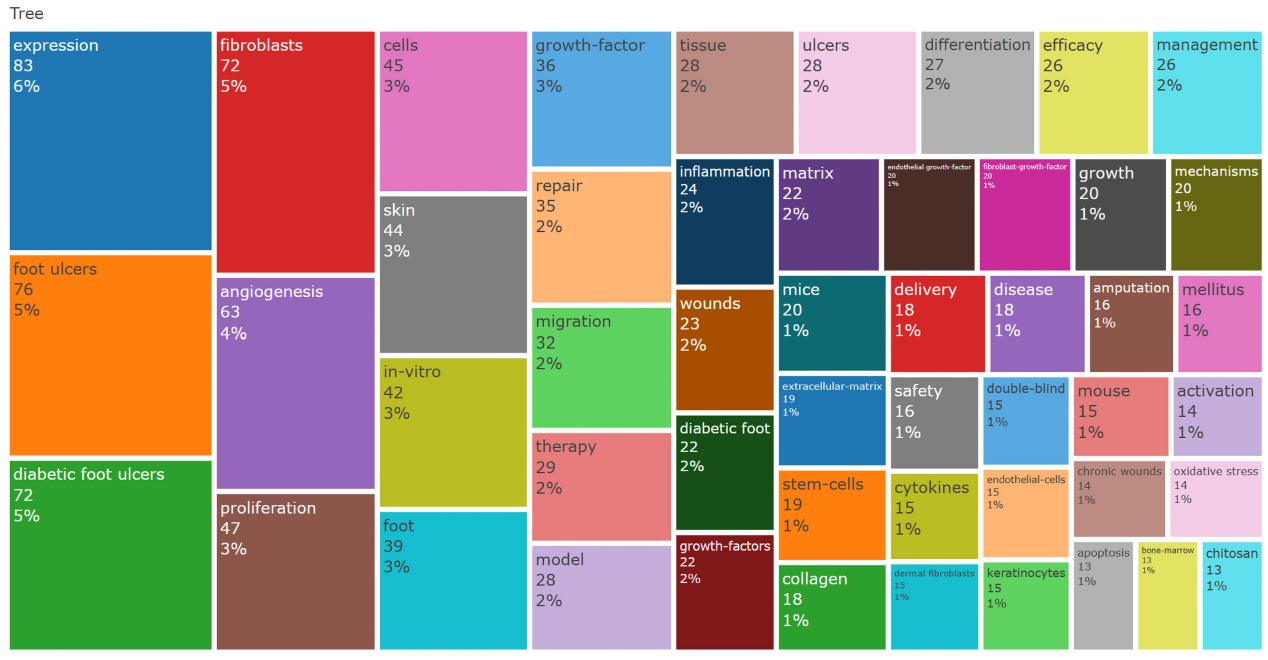


**Figure S5.** Keywords analysis. Visualized tree map based on the top 50 most frequent keywords for fibroblast-related DFUs research.


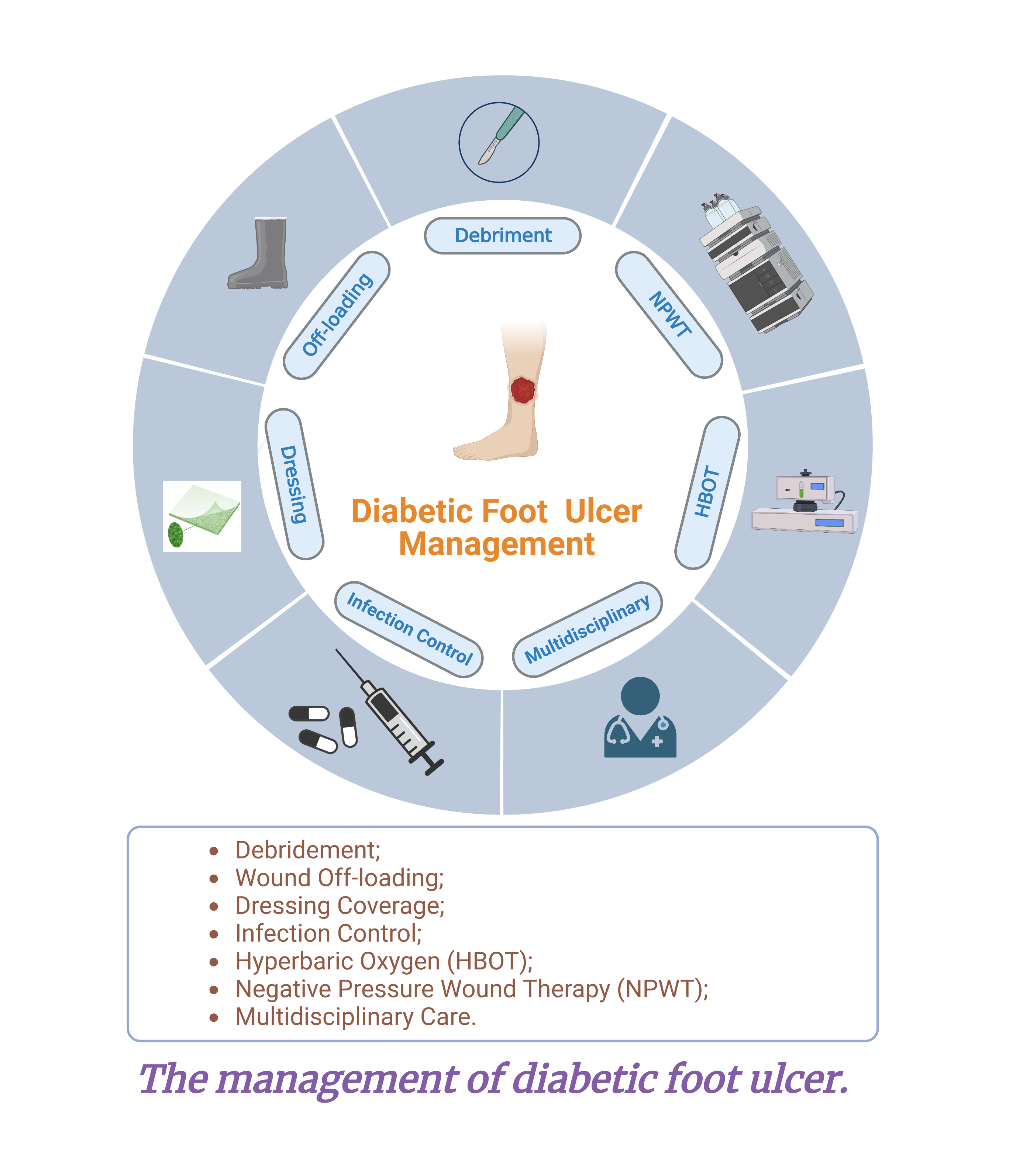


**Figure S6.** The management of diabetic foot ulcer. In the late 19th century, Treves established three essential principles for treating foot ulcers: rapid debridement, offloading pressure, and foot care. Subsequently, these principles laid a solid foundation to today's standard of care for DFUs: surgical debridement, wound off-loading, dressing coverage, and infection control. In addition, with the development of science and technology, various adjunctive therapies are used in the treatment of DFU, including hyperbaric oxygen and negative pressure wound therapy. More importantly, multidisciplinary diabetic foot care is becoming a focal point of treatment.
